# HSP70-1 is required for interleukin-5-induced angiogenic responses through eNOS pathway

**DOI:** 10.1038/srep44687

**Published:** 2017-03-20

**Authors:** Sung Lyea Park, Tae-Wook Chung, Sangtae Kim, Byungdoo Hwang, Jung Min Kim, Hwan Myung Lee, Hee-Jae Cha, Yoonhee Seo, Soo Young Choe, Ki-Tae Ha, Gonhyung Kim, Seok-Joong Yun, Sung-Soo Park, Yung Hyun Choi, Bo Kyung Kim, Won-Tae Kim, Eun-Jong Cha, Cam Patterson, Wun-Jae Kim, Sung-Kwon Moon

**Affiliations:** 1 Department of Food and Nutrition, Chung-Ang University, Anseong 456-756, Korea; 2 School of Korean Medicine and Healthy Aging Korean Medicine Research Center, Pusan National University, Yangsan, 626-780, Republic of Korea; 3 Biomedical Research Institute, Bundang Hospital, Seoul National University, 647-707, Korea; 4 NAR Center, Inc., Daejeon Oriental Hospital of Daejeon University, Daejeon 301-724, Republic of Korea; 5 Department of Cosmetic Science, Hoseo University, Asan-si 31499, Republic of Korea; 6 Department of Parasitology and Genetics, Kosin University College of Medicine, Busan 602-702, Republic of Korea; 7 EBO Co., Ltd., Future Convergence Technology Center, Cheongwon-Gun, Chungbuk, Korea; 8 Department of Biology, Chungbuk National University, Cheongju, Chungbuk, Korea; 9 Department of Veterinary Surgery, College of Veterinary Medicine, Chungbuk National University, Cheongju, Chungbuk, Korea; 10 Personalized Tumor Engineering Research Center, Department of Urology, Chungbuk National University, Cheongju, Chungbuk 361-763, South Korea; 11 Department of Food Science and Nutrition, Jeju National University, Jeju, Jeju Special Self-Governing Province, 690-756, South Korea; 12 Department of Biochemistry, College of Oriental Medicine, Dongeui University, Busan 614-052, South Korea; 13 Department of Physiology, College of Medicine, Konkuk University, Seoul 143-701, Republic of Korea; 14 Department of Biomedical Engineering, Chungbuk National University, Cheongju 361-763, Korea; 15 NewYork-Presbyterian Hospital, New York, NY 10065, USA

## Abstract

We report a pivotal role for IL-5 as an angiogenic activator. IL-5 increased proliferation, migration and colony tube formation in HUVECs associated with the phosphorylation of ERK and AKT/eNOS, and promoted microvessel sprouting from an angiogenesis animal model. The angiogenic effects were confirmed in IL-5-deficient mice and addition of IL-5 antibody. HSP70-1 was identified via expression profiling following IL-5 stimulation. A siRNA knockdown of HSP70-1 suppressed angiogenic responses and eNOS phosphorylation induced by IL-5. HSP70-1 overexpression enhanced IL-5-induced angiogenic responses. In addition, IL-5-induced neo-vascular formation was verified in both HSP70-1 knockout and HSP70-1 transgenic mice. Furthermore, transcription factor AP-1 was a main factor in IL-5-induced HSP70-1 in response to ERK and AKT signaling pathway. Angiogenic responses induced by VEGF had no effect in either HSP70-1 siRNA *in vitro* or HSP70-1 knockout mice. IL-5-induced angiogenic responses depended on the binding of IL-5Rα. Our data demonstrate that binding of IL-5 to IL-5Rα receptors enhances angiogenic responses by stimulating the expression of HSP70-1 via the eNOS signaling pathway.

Angiogenesis, the formation of new blood vessels, plays a crucial role in the progression of both tumor development and inflammatory diseases^1^. The elaborate network between pro-angiogenic factors and the microenvironment regulates the induction of new blood vessel formation^2^,^3^. The cytokines and growth factors that are considered to be angiogenic factors include VEGF, TGF-β1, PDGF, IL-8, and bFGF that are produced by endothelial cells, as well as inflammatory cells and tumor cells^1^,^2^,^3^. Angiogenesis is believed to play a primary role in the proliferation, migration, invasion, and tube formation of endothelial cells^1^,^2^.

Interleukin-5 (IL-5) is a cytokine that is produced by activated T cells, and is referred to as a T-cell replacing factor (TRF)^4^,^5^. IL-5 has exhibited a wide range of biological functions, such as the differentiation of B-cells and the production and proliferation of eosinophils^4^,^5^. IL-5 initiates a signal via a heterodimeric receptor, which consists of a ligand-specific IL-5Rα and a common βc^4^,^5^. IL-5 induces the proliferation of B cells, which are involved in the activation of PI3K, Shc and JaK2^5^. Previous studies have demonstrated that the activation of MAPK, Syk, Jak2/Stat1, and PI3 kinase is critical for the regulation of eosinophils^5^. Several essential enhancer element-binding sites, such as c/EBPβ, Sp1, Oct-2 and E12/E47, have been identified in B cells and eosinophils^6^. Recent study from our group showed that IL-5 induces the migration and invasion of bladder cancer cells^7^,^8^. However, little is known about the functional roles of IL-5 in the angiogenic responses.

Heat-shock proteins (HSPs) are expressed constitutively and are induced by various types of stimuli that include hypoxia, heat shock, glucose deprivation, and ischemia^9^. Members of the 70-kDa HSP (HSP70) family are among the most extensively studied molecular chaperones; they aid in protein folding, synthesis, assembly, and trafficking between cellular compartments^10^,^11^. The HSP70 family consists of eight homologous molecular chaperones^12^. Among them, the expressions of HSP70-1 and HSP70-3 are induced in response to environmental stress, which protects the cells that are exposed to lethal damage^13^. In addition, the expression of HSP70-2 is highly expressed during the meiotic phase of spermatogenesis^12^. HSP70-1 disruption in mice increases infarction volume after acute focal cerebral ischemia and allows for sensitization to osmotic stress^14^,^15^. A deficiency in HSP70-2 results in failed meiosis, germ cell apoptosis, and male infertility^16^. However, the role of HSP70-1 in angiogenesis has not yet been studied.

In the present study, we tested a novel theory wherein inflammatory cytokine IL-5 could induce angiogenic responses via the binding of its receptor, IL-5Rα. Furthermore, in additional study using *in vitro* and genetic mice models, the molecular chaperone HSP70-1 played a pivotal role in mediating the pro-angiogenic effects of IL-5.

## Results

### IL-5 induces angiogenic responses via phosphorylation of ERK1/2 and AKT/eNOS in HUVECs

**Figure 1:**
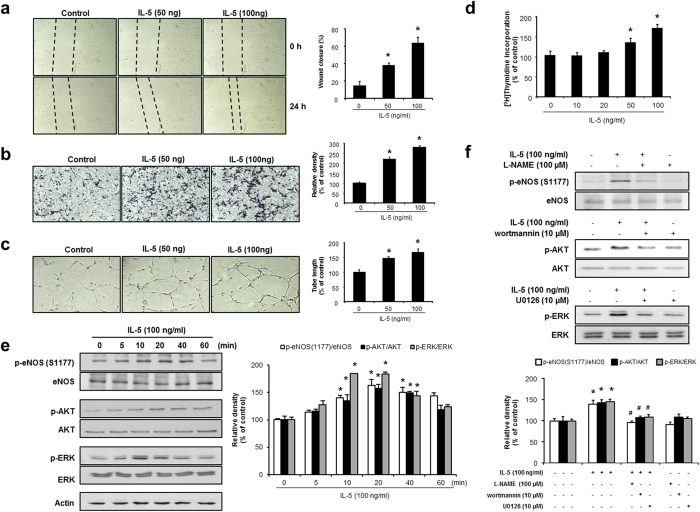
IL-5 induced the proliferation, migration, and colony tube formation and phosphorylation of ERK1/2 and AKT/eNOS in HUVECs. (**a**) Wound-healing assays revealed a significant induction in the wound closure rates of HUVECs after IL-5 stimulation for 24 h. (**b**) Invasion assay of HUVECs induced by IL-5 for 24 h. (**c**) Induction of capillary tube formation in IL-5-treated HUVECs. (**d**) Proliferative effect of IL-5 in HUVECs as evaluated by [^3^H]thymidine incorporation assay. (**e**,**f**) Phosphorylation of ERK1/2, AKT, and eNOS (S1177) in the presence or absence of U0126, wortmannin, and L-NAME, followed by IL-5 treated with HUVECs. All data are reported as the means ± SE from three independent experiments. *P < 0.05 compared with control, ^#^*P* < 0.05 compared with IL-5 treatment.

To first investigate the angiogenic responses of IL-5 *in vitro*, we evaluated the proliferation, migration, and invasion of HUVECs using a [^3^H]thymidine incorporation assay, a wound-healing assay, and a Boyden Chamber assay, respectively. IL-5 significantly increased the proliferation, wound closure rate, and invasiveness of HUVECs (Fig. 1a,b,d). Next, we determined the effect of IL-5 on the morphologic organization of 2-dimensional colony tube formation in HUVECs. Treatment of IL-5 with HUVECs significantly extended the colony tube length compared with non-treatment (control) (Fig. 1c). In addition, control did not induce the angiogenic responses, although HUVECs showed the endogenous expression of IL-5 (Fig. S1a), demonstrating that IL-5 mediates a paracrine mechanism of HUVECs. To identify the regulatory mechanism of IL-5 in angiogenesis, we examined the signaling pathways using specific antibodies to ERK1/2, AKT, and eNOS. As shown in Fig. 1e, treatment of IL-5 with HUVECs induced the phosphorylation of ERK1/2, AKT, and eNOS. The phosphorylation of ERK1/2, AKT, and eNOS was inhibited by its specific inhibitors U0126, wortmannin and L-NAME in HUVECs (Fig. 1f). Furthermore, blockage of ERK1/2, AKT, and eNOS pathway suppressed the angiogenic responses in response to IL-5 (Fig. S2). Finally, we confirmed increased endothelial nitric oxide (NO) production in IL-5-treated HUVECs compared with control (Fig. S12a,b).

### IL-5 stimulates angiogenesis *ex vivo* and *in vivo*

**Figure 2:**
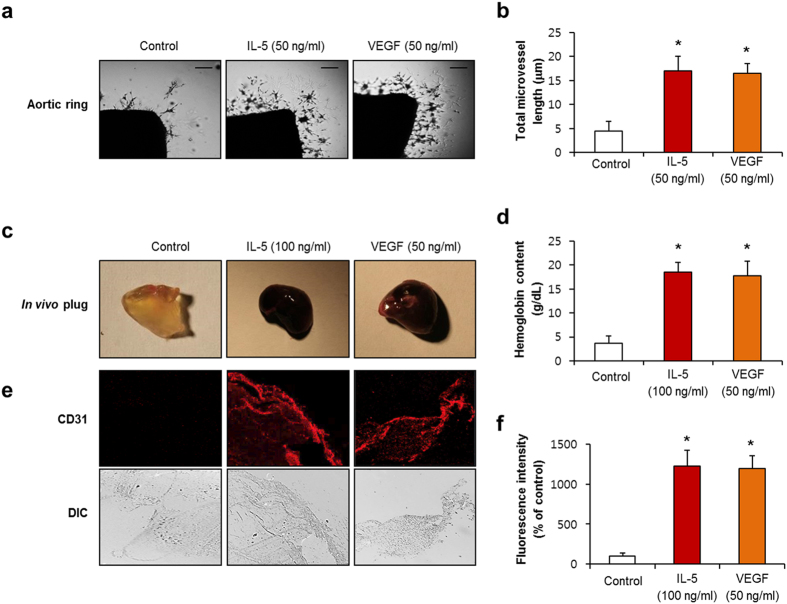
IL-5 induced microvessel formation of angiogenesis *ex vivo* and *in vivo*. (**a**) Angiogenesis *ex vivo* determined by aortic ring assay at 9 days. (**b**) The number of neo-vessel sprouts in an aortic ring assay. (**c**) Photographs of the matrigel plug *in vivo* assay at 7 days. (**d**) Quantitative analysis of neovessel formation by hemoglobin contents in the matrigel. (**e**) Immunostaining of the matrigel plug with CD31 antibody. (**f**) Quantification of the area of CD31-positive vessels. All data are reported as the means ± SE from three independent experiments. *P < 0.05 compared with control.

To investigate the effect of IL-5 in angiogenesis in an *ex vivo* animal model, an aortic ring assay was employed. IL-5 (50 ng/ml) treatment promoted the total number of sprouting microvessels that emerged from aortic rings compared with the controls (Fig. 2a,b). To further obtain *in vivo* evidence for IL-5-mediated angiogenesis, a matrigel plug *in vivo* assay was next conducted. A matrigel plug containing IL-5 was filled with a dark red color compared with the control, which corresponded to the results of neovessel formation (Fig. 2c). The hemoglobin content in the matrigel plug treated with IL-5 was about 4.4-fold higher than that of the control (Fig. 2d). This new vessel formation of the capillary was confirmed using immunostaining of the endothelial specific marker CD31 (Fig. 2e,f). In addition, IL-5-induced angiogenic responses were confirmed by the use of neutralizing antibody for IL-5 (Fig. S3). Furthermore, plasma NO production was increased in the matrigel plug injected with IL-5 compared with control (Fig. S12c). These results strongly support the angiogenesis capacity of IL-5 under both *ex vivo* and *in vivo* conditions.

### Effects of angiogenesis on IL-5-deficient mice

**Figure 3:**
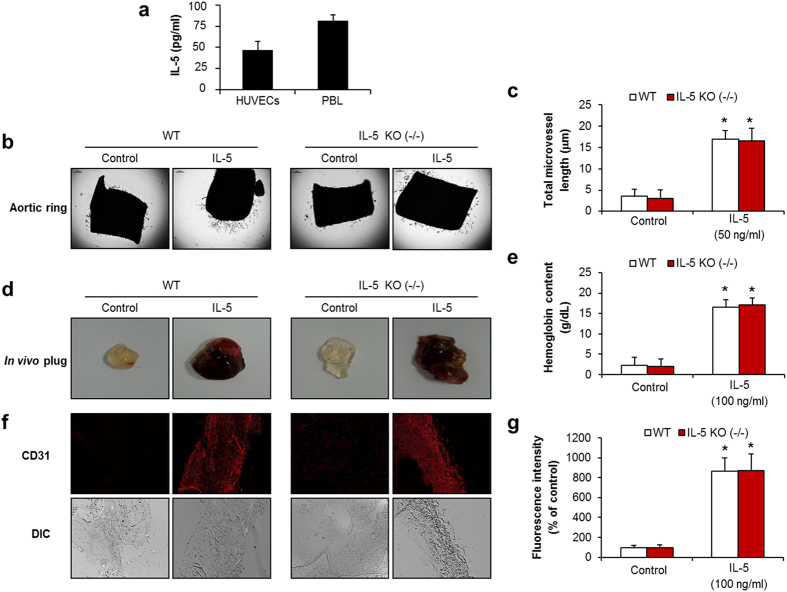
The neo-microvessel formation of angiogenesis in IL-5-deficient mice. (**a**) ELISA immunoassay of IL-5 in cultured HUVECs. (**b**) Aortic ring assay in IL-5-deficient mice at 9 days. (**c**) Quantification of aortic ring sprouting. (**d**) Images of matrigel plug angiogenesis assay at 7 days. (**e**) Determination of hemoglobin contents in the matrigel plug. (**f**) CD31 staining in matrigel plugs. (**g**) The area of CD31-positive vessels was quantified. All data are reported as the means ± SE from three independent experiments. *P < 0.05 compared with control.

We performed an ELISA assay for IL-5 in conditioned medium from HUVECs. HUVECs produced about 47 pg/ml of low-level IL-5 protein in conditioned medium (Fig. 3a). These results suggest that low-level IL-5 protein could not induce the angiogenic responses under normal conditions in HUVECs. We next investigated how the lack of IL-5 would affect the physiological angiogenesis of IL-5-deficient mice. We found no differences in the microvessel sprouting from IL-5-deficient mice compared with WT groups in an aortic ring assay (Fig. 3b,c). However, IL-5 treatment induced microvessel sprouting from aortic ring in IL-5-deficient mice (Fig. 3b,c). The increased levels of microvessel outgrowth that were induced by IL-5 were equivalent for both the IL-5 deficient mice and the WT groups (Fig. 3b,c). Similar results were observed in an *in vivo* plug assay (Fig. 3d–g). These results support the strong angiogenesis effect of IL-5 as a paracrine factor.

### Gene expression profile in IL-5-treated HUVECs

**Figure 4:**
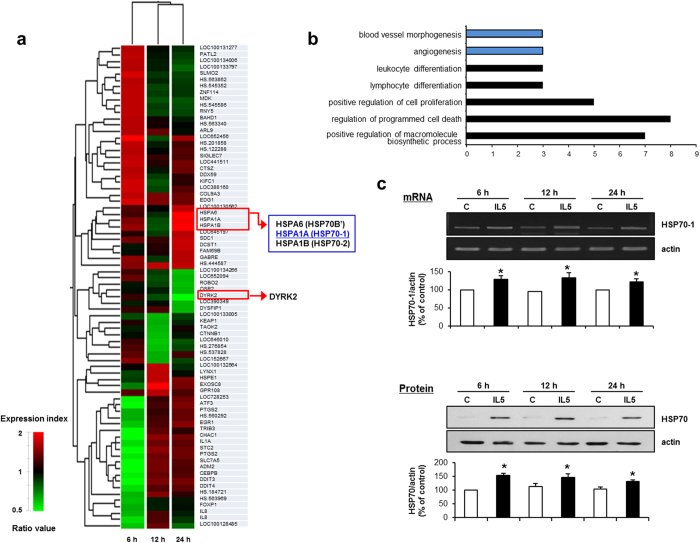
Gene expression patterns of IL-5-induced target genes in HUVECs. (**a**) Hierarchical clustering of IL5-induced genes. A gene set representing >1.5-fold changes in at least one time point is presented by hierarchical clustering (HC) analysis (red, >1.5-fold change; green, <1.5-fold change). The HC structure (dendrogram) representing similarities in the expression patterns between experimental samples. The clustering is represented using tools provided by GeneSpring GX 7.3 software (Agilent Technologies, Santa Clara, CA, USA). (**b**) The results of Gene Ontology analysis by microarray approaches in response to IL-5. Gene lists corresponding to the 1.5-fold up- and down-regulation in the IL-5-treated HUVEC cells are constructed using DAVID for Gene Ontology analysis (http://david.abcc.ncifcrf.gov/). (**c**) IL-5-induced expression of HSP70-1 was performed by RT-PCR and immunoblot at the indicated time points. All data are reported as the means ± SE from three independent experiments. *P < 0.05 compared with control.

To elucidate the IL-5-dependent expression patterns of mRNA, HUVECs were treated with or without IL-5 for 6 h, 12 h, and 24 h. The differentially expressed gene expression patterns were characterized by microarray analysis. Thirty-six (36) genes showed up-regulated expressions and forty-one (41) genes were down-regulated at any time point in the IL-5-treated HUVECs, compared with the control cells (Table S1). Both hierarchical clustering analysis and gene ontology analysis of the gene expression patterns were employed to identify the molecular characteristics between the two groups (Fig. 4a,b). The results from those analyses revealed the biological functions associated with functional categories (Fig. 4b). Our data demonstrated that genes were significantly enriched when they were involved in the regulation of programmed cell death, the positive regulation of the macromolecule biosynthetic process, cell proliferation, blood vessel morphogenesis, angiogenesis, leukocyte differentiation, and lymphocyte differentiation (Fig. 4b). The greatest fold-change of up-regulated genes by IL-5 occurred with heat-shock proteins, HSP70-1, HSP70-2, and HSPA6 at 24 h (Fig. 4a and Table S1). In addition, DYRK2 was identified as the most highly down-regulated gene in IL-5-treated HUVECs at 24 h (Fig. 4a and Table S1). Because HSP70-2 knockout mice experienced pathological dysfunctions such as male infertility^16^ and HSPA6 expression that are normally observed only in humans, swine and goats^17^, we finally selected a HSP70-1 to determine the angiogenic responses in IL-5-treated HUVECs.

### siRNA-mediated inhibition of HSP70-1 suppresses IL-5-mediated proliferation, wound-healing migration, invasion, and colony tube formation in HUVECs

**Figure 5:**
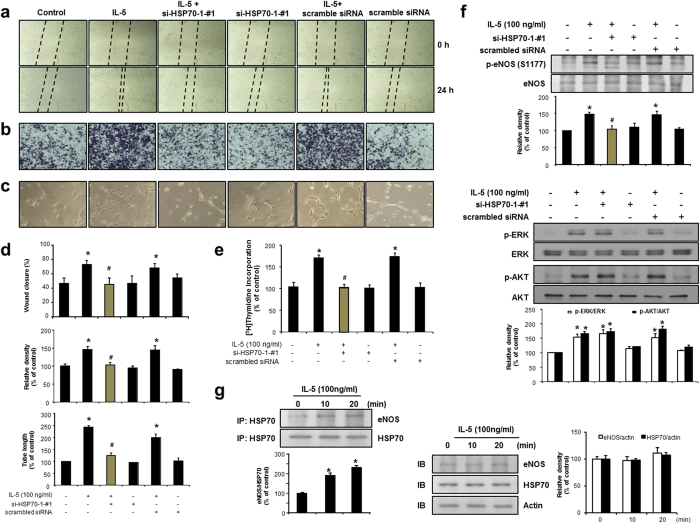
siRNA-mediated knockdown of HSP70-1 led to the dysfunction of IL-5-induced proliferation, migration, colony tube formation, and phosphorylation of eNOS in HUVECs. (**a**–**e**) IL-5-induced proliferation, migration, invasion, and colony tube formation was determined in HSP70-1 siRNA (si-HSP70-1-#1) transfected HUVECs. (**f**) After transfection of HSP70-1 siRNA (si-HSP70-1-#1) or scrambled siRNA for 24 h, cells were subjected to immunoblot analysis using specific antibodies for ERK1/2, AKT, and eNOS (S1177). (**g**) Serum-starved HUVECs were incubated with IL-5 for indicated times. Equal amounts of cell lysates were immunoprecipitated with anti-HSP70 antibody and analyzed by immunoblot using antibodies specific for eNOS and actin. All data are reported as the means ± SE from three independent experiments. *P < 0.05 compared with control, ^#^*P* < 0.05 compared with IL-5 treatment.

Because HSP70-1 was identified as having the greatest fold-change of expression from microarray analysis in IL-5-treated HUVECs, we next validated the expression of HSP70-1 at 6 h, 12 h, and 24 h using RT-PCR analysis. The up-regulated expression level of HSP70-1 was found in HUVECs followed by IL-5 at the mRNA level (Fig. 4c). In addition, the level of induction for HSP70-1 protein was confirmed by immunoblot analysis using a HSP70 antibody^14^, which had a HSP70-1 specific binding epitope (Fig. 4c). To further investigate the role of HSP70-1 in IL-5-induced angiogenic responses, we used three types of siRNAs specific for HSP70-1: a HSP70-1-specific siRNA (si-HSP70-1-#1 and -#2) or a scrambled siRNA (scramble) (Fig. S1b). The proliferation, wound-healing migration and invasion of HUVECs induced by IL-5 were suppressed in si-HSP70-1-transfected HUVECs compared with the scrambled version (Fig. 5a,b,e and Fig. S4a,b,e). Moreover, transfection of si-HSP70-1 significantly blocked the IL-5-induced colony tube formation of HUVECs (Fig. 5c and Fig. S4c). These results demonstrate that IL-5 induces proliferation, wound-healing migration, invasion and colony tube formation of HUVECs through HSP70-1 expression.

### IL-5 stimulates phosphorylation of eNOS via HSP70-1 expression in HUVECs

To test whether HSP70-1 affects the IL-5-mediated signaling pathway in HUVECs, the phosphorylation of ERK1/2, AKT, and eNOS was examined. A blockade of the HSP70-1 gene inhibited the IL-5-induced eNOS phosphorylation in HUVECs (Fig. 5f and Fig. S4f). However, the phosphorylation of ERK1/2 and AKT had no effect in the presence of the si-HSP70-1 gene (Fig. 5f and Fig. S4f). Next, we examined whether HSP70-1 could interact with eNOS in IL-5-treated HUVECs. In immunoprecipitation experimentation, IL-5 treatment resulted in a significant increase in the interaction of HSP70-1 and eNOS in HUVECs (Fig. 5g). These findings indicate that HSP70-1 facilitates eNOS signaling when mediated by IL-5, and that it leads to angiogenic responses in HUVECs.

### HSP70-1 expression is required for IL-5-induced angiogenesis *ex vivo* and *in vivo*

**Figure 6:**
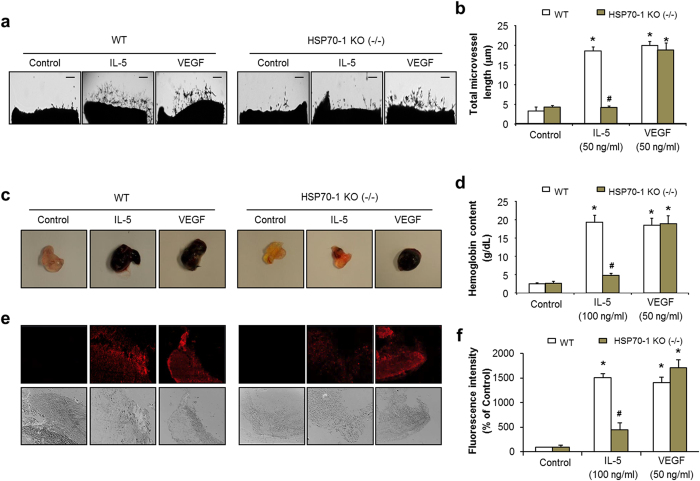
Dysfunction of IL-5-induced angiogenesis *ex vivo* and *in vivo* in HSP70-1 knockout mice (KO). (**a**) Aortic ring assay was performed in wild type (WT) and HSP70-1 knockout mice (KO) at 9 days. (**b**) Quantitative analysis of microvessel sprouting in aortic rings. (**c**) Matrigel plug *in vivo* assay in wild type (WT) and HSP70-1 knockout mice (KO) at 7 days. (**d**) Quantification of hemoglobin contents. (**e**) The matrigel plugs were immunostained with antibody against CD31. (**f**) Determination of the area of CD31-positive vessels. All data are reported as the means ± SE from three independent experiments. *P < 0.05 compared with control, ^#^*P* < 0.05 compared with IL-5 treatment in WT.

A genetic deletion mice model of HSP70-1 was used to further elucidate the direct evidence of the HSP70-1 involvement in IL-5-induced angiogenesis. As shown in Fig. 6a and b, the outgrowth of microvessel formation by IL-5 was blocked in aortas from HSP70-1(−/−) mice models. In addition, we found that the neovessel formation of matrigel implantation induced by IL-5 was substantially impaired in the HSP70-1(−/−) mice compared with wild-type mice (Fig. 6c,d). These results were confirmed by immunostaining analysis of the endothelial cell specific marker CD31 (Fig. 6e,f). However, it is interesting, that the VEGF-mediated neovascularization formed by aortic rings and matrigel plugs was significantly increased in both wild-type and HSP70-1(−/−) mice (Fig. 6a–f). Moreover, IL-5 treatment did not induce expression levels of VEGF-C, VEGFR, or VEGFR phosphorylation in HUVECs (Fig. S1c,d). These results suggest that HSP70-1 expression is essential for IL-5-mediated angiogenic responses, but that it is independent of VEGF. Our data also revealed that the expression level of angiogenic growth factors such as VEGF-A, bFGF, Angiopoietin-1, and Angiopoietin-2 was not observed in IL-5-treated HUVECs for neither mRNA nor protein level (Fig. S5a,b).

### AP-1 is associated with IL-5-induced HSP70-1 expression via ERK1/2 and AKT signaling

IL-5 stimulated phosphorylation of AKT and ERK1/2 (Fig. 1e). Inhibition of AKT and ERK1/2 signaling suppressed IL-5-induced angiogenic responses (Fig. S2a–g). We next examined the linkage between the signaling pathways and HSP70-1 expression in IL-5-treated HUVECs. We found that the inhibition of AKT and ERK1/2 using wortmannin and U0126 blocked the HSP70-1 expression induced by IL-5 (Fig. S6a). In general, cytokine IL-5 regulate the gene expression through elevation of transcription factors such as NF-κB, AP-1 and Sp-1^5^,^7^,^8^. Bioinformatics analysis using promo search software (Version 3) revealed that the promoter region of the human HSP70-1 gene contains the putative DNA-binding sites of AP-1 and Sp-1. Subsequently, EMSA experiment was conducted to examine the transcriptional regulation in IL-5-induced HSP70-1. Addition of IL-5 induced AP-1 binding activity in HUVECs at indicated times (Fig. S6b). However, no specific binding complexes to Sp-1 were detected in HUVECs treated with IL-5 (Fig. S6b). These results indicate that transcription factor AP-1 is involved in the IL-5-induced HSP70-1 expression. To further investigate the role of signaling pathways in the transcriptional regulation correlated with the increased HSP70-1 expression in IL-5-stimulated HUVECs, we performed an EMSA using AP-1 motifs. IL-5-induced AP-1 binding activity was significantly suppressed by the addition of U0126 and wortmannin in HUVECs (Fig. S6c). These findings demonstrate that AP-1 might be an important factor associated with signaling pathways–mediated control of HSP70-1 expression in IL-5-stimulated angiogenic responses.

### HSP70-1 expression is not involved in VEGF-mediated angiogenic responses in HUVECs

Because VEGF-mediated angiogenesis was not inhibited in the HSP70-1 knockout mice model (Fig. 6), we next investigated the role of HSP70-1 in VEGF-induced angiogenic responses in HUVECs. The knockdown of HSP70-1 did not affect the proliferation, migration, invasion, and colony tube formation of HUVECs induced by VEGF (Figs S7a–d and S8a–d). Furthermore, the VEGF-mediated phosphorylation of eNOS was not suppressed by the transfection of HSP70-1 siRNA (Figs S7e and S8e). These findings demonstrate that HSP70-1 may not be associated with VEGF-mediated angiogenic responses.

### Overexpression of HSP70-1 enhances IL-5-induced angiogenesis *in vitro, ex vivo*, and *in vivo*

**Figure 7:**
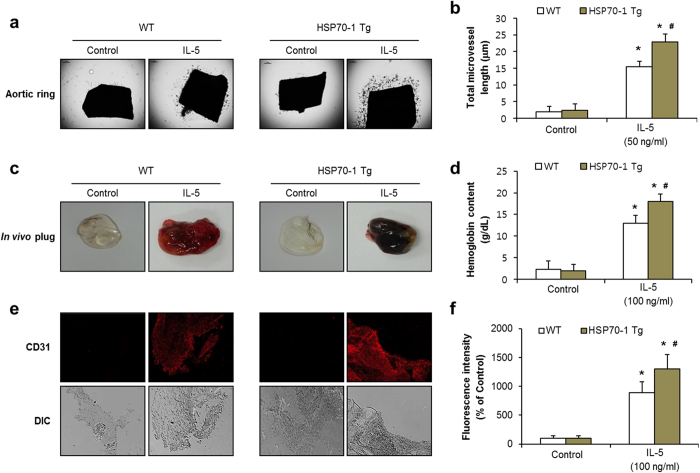
IL-5-induced angiogenesis is enhanced in HSP70-1-transgenic mice. (**a**) Aortic ring assay in wild type (WT) and HSP70-1-transgenic mice at 5 days (Tg). (**b**) Statistical analysis of neo-vessel sprouting in aortic rings. (**c**) Matrigel plug *in vivo* images in wild type (WT) and HSP70-1-transgenic mice (Tg) at 4 days. (**d**) Quantitative assessment of hemoglobin contents. (**e**) Immunostaining of CD31 in matrigel plugs. (**f**) Statistical results of the area of CD31-positive vessels. All data are reported as the means ± SE from three independent experiments. *P < 0.05 compared with control, ^#^*P* < 0.05 compared with IL-5 treatment in WT.

We next investigated whether HSP70-1 overexpression affects IL-5-induced angiogenic responses. Overexpression of the HSP70-1 gene did not induce angiogenic responses in HUVECs (Fig. S10a–g). However, the IL-5-induced proliferation, migration, invasion, and colony tube formation of HUVECs were slightly up-regulated via transfection of the HSP70-1 gene (Fig. S10a–g). To confirm the effect of HSP70-1 overexpression in both *ex vivo* and *in vivo* angiogenic responses, we employed HSP70-1 transgenic mice. To this end, we examined the outgrowth of microvessel sprouting and neovessel formation of matrigel implantation at short time period (Fig. 7a–f). The increased outgrowth of microvessel formation from the aortic ring was not observed in the transgenic overexpression of HSP70-1 compared with WT mice at 5 days (Fig. 7a,b). The neovessel formation of matrigel plug was similar in both the transgenic and the WT group at 4 days (Fig. 7c–f). Interestingly, IL-5-induced angiogenic responses in both *ex vivo* and *in vivo* were significantly enhanced in HSP70-1 transgenic mice compared with WT mice (Fig. 7a–f). These data indicate that HSP70-1 overexpression mediates the angiogenic effects in response to IL-5.

### IL-5 induces proliferation, migration, invasion, colony tube formation, and eNOS phosphorylation via IL-5Rα in HUVECs

**Figure 8:**
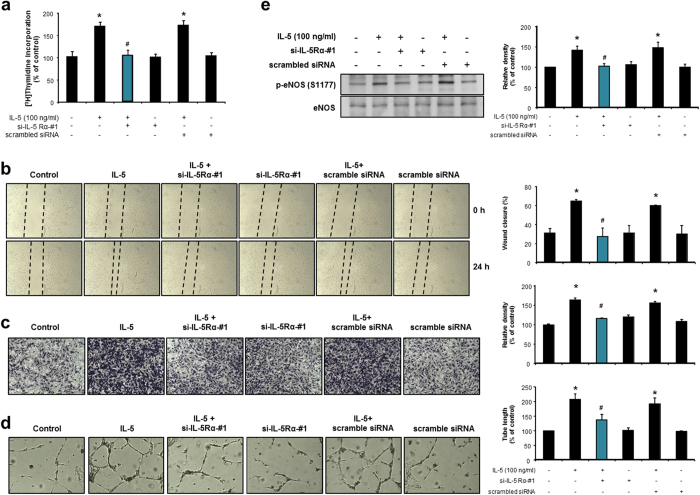
IL-5 induced angiogenic responses via binding of IL-5Rα in HUVECs. (**a–d**) After transfection of IL-5Rα siRNA (si-IL-5Rα-#1) or scrambled siRNA for 24 h, cells were analyzed to determine the IL-5-stimulated proliferation, migration, invasion, and colony tube formation. (**e**) Immunoblot of phospho-eNOS (S1177) and eNOS in the IL-5Rα siRNA (si-IL-5Rα-#1) or scrambled siRNA transfected cells. All data are reported as the means ± SE from three independent experiments. *P < 0.05 compared with control, ^#^*P* < 0.05 compared with IL-5 treatment.

Subsequently, we observed the increased expression of IL-5Rα in IL-5-treated HUVECs (Fig. S1e). To determine whether IL-5 can transduce angiogenic responses via its receptor IL-5Rα, the potential role of IL-5 in HUVECs was further investigated using a ligand-specific receptor in an IL-5Rα specific siRNA system (si-IL-5Rα-#1 and -#2) (Fig. S1f). As shown in Fig. 8a–d and Fig. S9a-d, the knockdown of IL-5Rα abolished the increased proliferation, migration, invasion, and colony tube formation of HUVECs in response to IL-5, compared with control cells or scrambled siRNA transfected cells. Moreover, si-IL-5Rα siRNA effectively blocked the IL-5-induced phosphorylation of eNOS in HUVECs (Figs 8e and S9e). These data demonstrate that IL-5 induces angiogenic responses and eNOS phosphorylation via the binding of IL-5Rα in HUVECs. We next investigated whether overexpression of HSP70-1 rescues the effect of IL-5Rα-silencing on IL-5-induced endothelial cell proliferation, migration, invasion, and tube formation. HSP70-1 overexpression could not rescue the decreased IL-5-induced endothelial cell proliferation, migration, invasion, and tube formation in the presence of si-IL-5Rα (Fig. S10a–g), demonstrating that HSP70-1 would not affect the angiogenic effects in the absence of IL-5. In addition, we also examined the effect of eNOS gene on proliferation, migration, invasion, and tube formation of endothelial cells induced by IL-5 in the presence of HSP70-1 silencing. Overexpression of the eNOS gene induced the migration and invasion of HUVECs (Fig. S11a,b,d,e). However, eNOS overexpression changed neither the proliferation nor the tube formation of HUVECs (Fig. S11c,f,g). In addition, overexpression of eNOS could not reverse the effect of HSP70-1 silencing on IL-5-induced endothelial cell proliferation, migration, invasion, and tube formation (Fig. S11a–g). These results suggest that eNOS alone was not sufficient to affect the IL-5-induced proliferation, migration, invasion, and tube formation of HUVECs.

## Discussion

Inflammatory-mediated vascular injury that is deeply relevant to rheumatoid arthritis, various forms of cancer, and atherosclerosis has attracted a great deal of attention. Little is known, however, about the exact roles and mechanisms of inflammatory cytokines in mediating angiogenesis. In the present study, we demonstrated that IL-5 promotes angiogenic responses through its receptor, IL-5Rα, which was confirmed by IL-5 deficient mice and addition of IL-5 antibody. In addition, we used microarray analysis and siRNA technic to reveal the novel function of HSP70-1 that is involved in IL-5-induced angiogenesis. It is noteworthy that a deficiency of HSP70-1 in mice independently caused a significant impairment of the angiogenic responses induced by IL-5 upon the VEGF. Consistently, HSP70-1 overexpressing mice enhanced IL-5-induced angiogenesis. Furthermore, AP-1 was a key mediator in IL-5-induced HSP70-1 expression via ERK and AKT signaling pathways. These findings are evidence of inflammatory cytokine IL-5 involvement in vascular development.

The roles of IL-5 in the pathogenesis of asthma and allergic diseases have been extensively investigated. IL-5 is primarily produced by CD4+ Th2 cells with lower amounts secreted by activated eosinophils and mast cells^4^,^5^,^6^. Studies have focused on the proliferation and differentiation of B cells and eosinophils in response to IL-5^4^,^5^. Recent results from one of our studies showed that IL-5 induced the migration and invasion of bladder cancer cells^7^,^8^. In that report, we further demonstrated that ERK1/2 signaling and p21WAF1 expression is involved in the migration and invasion of bladder cancer cells induced by IL-5^7^,^8^. The role of IL-5 on HUVECs in the present study showed that IL-5 stimulated colony tube formation, proliferation, migration, and invasion of the cells. In addition, IL-5 increased the phosphorylation of S1177 of eNOS (eNOS activity by phosphorylation at Ser-1177) and the phosphorylation of both AKT and ERK1/2, as main signaling pathways involved in the induction of angiogenic endothelial cells, without altering the levels of VEGF-A, VEGF-C, bFGF, Angiopoietin-1, or Angiopoietin-2. Furthermore, IL-5 increased the promotion of vessel sprouting and neo-vasculature formation, as shown in aortic ring and *in vivo* matrigel plug assays. We also confirmed the increased NO production levels in HUVECs and matrigel plug mice plasma followed by addition of IL-5. Several signaling pathways, including the AKT/eNOS pathway, the MAPK pathway, and the integrins/FAK-mediated pathway, are activated during angiogenesis^18^,^19^,^20^. The signaling pathway of IL-5 in endothelial cells (EC) has not yet been reported. Our present results indicated that the binding of IL-5 to IL-5Rα induces angiogenic responses through the eNOS pathway. Some studies have reported that inflammatory cytokines, such as IL-1β, IL-8, IL-32, and IL-33, function as angiogenic factors^2^,^20^,^21^. These cytokines have shown dual angiogenic action by inducing EC responses directly or by recruiting inflammatory cells that indirectly leads to the release of EC mitogen^2^,^20^,^21^,^22^. In the present study, the endogenous low level of IL-5 produced by HUVECs affected neither angiogenic function nor signaling. The addition of exogenous recombinant IL-5 induced the angiogenic effect and signaling. IL-5-induced angiogenic responses were reversed by addition of IL-5 antibody. This implies that IL-5 released by inflammatory cells acts as a selective regulator for angiogenic function and signaling. These results suggest that IL-5 functions predominately in a paracrine manner for angiogenesis.

In the current study, there was no evidence of angiogenesis in either WT or IL-5 knockout mice. IL-5 treatment induced angiogenesis in IL-5 knockout mice, and the increased level of angiogenesis in IL-5-deficient mice was similar to that of WT mice, indicating a paracrine role for the IL-5. During several pathophysiological conditions, IL-5 is produced by inflammatory cells including activated T-cells, mast cells, and eosinophil cells^4^,^5^,^6^. It is important to determine whether the presence of IL-5 in matrigel plugs affects infiltration and/or activation of inflammatory cells known to respond to IL-5. However, we could not analyze the infiltration of inflammatory cells to the matrigel plug due to limitations in our experimental system. Considering previous study demonstrating that IL-5 treatment increased infiltration of lungs with eosinophils using IL-5-deficient mice^23^, we could indirectly assume the possibility of infiltration and/or activation of inflammatory cells in response to IL-5. Therefore, additional studies are required to elucidate the infiltration of inflammatory cells that contribute to the promotion of IL-5-mediated angiogenesis.

Based on the present microarray dataset, we have established a comprehensive list of IL-5 target genes in HUVECs. Thirty-six (36) genes were the most highly expressed in IL-5-treated HUVECs. These genes also were classified into seven (7) major clusters in IL-5-rsponsive gene expression patterns using gene ontology analysis, suggesting that the genes with at least a 1.5-fold change in up-regulated expression were involved in the biological regulatory systems that are required for immunological or inflammatory-associated cytokine IL-5-induced angiogenesis signaling. Subsequently, to assess the role and molecular mechanism of novel target genes in IL-5-induced angiogenic responses, we focused on genes encoding heat shock protein such as HSP70-1, HSP70-2, and HSPA6 that encode a heat shock protein because the expression levels of those genes were the most highly up-regulated genes in response to IL-5 in HUVECs.

HSP70-1 was originally found as a molecular chaperone with a role in cell protection that was induced by various external insults^13^,^14^,^15^,^24^,^25^. Several studies have demonstrated animal experiments using HSP70-1 knockout or transgenic mice that are resistant to UVB irradiation and are involved in cardiac dysfunction and neuro-protective effects after ischemia/reperfusion, acute focal cerebral ischemia, and spinal cord injury^14^,^15^,^24^,^25^,^26^,^27^. Because the HSP70-2-deficient mice led to the disruption of meiosis and the triggering of apoptosis in the germ cells during spermatogenesis^12^,^16^,^28^, we were unable to obtain sufficient HSP70-2 (−/−) mice. In addition, HSPA6 expression was only detected in humans, swine and goats^17^. Therefore, we focused on analyzing the role and function of HSP70-1 in the angiogenic process that is induced by IL-5 using a siRNA knockdown system, HSP70-1 deficient mice, HSP70-1 gene overexpression, and HSP70-1 transgenic mice. In terms of the stress conditions, many studies have demonstrated that molecular chaperons, such as HSP16, mtHSP70, HSP27, and HSP70, act to control cell survival and apoptosis^9^,^11^. The involvement of HSPA12B has been demonstrated in the promotion of myocardial angiogenesis^29^. HSP70 rescued the Akt-FOXO3a-induced apoptosis of EC^30^. A double knockout of HSP70-1 (−/−) and HSP70-2 (−/−) resulted in the induction of cardiac dysfunction after ischemia/reperfusion via activation of JNK, p38-MAPK, Raf-1, and ERK^31^. HSP90 was implicated in the induction of angiogenesis mediated by AKT/eNOS signaling^32^. One report has suggested that HSC70 regulated the VEGF-induced EC function via AKT phosphorylation^33^. Despite the obviously critical role of molecular chaperones during angiogenesis^34^,^35^,^36^, no available information exists regarding the relationship between molecular chaperones and inflammatory cytokines in the promotion of angiogenesis. Our data from the siRNA knockdown of HSP70-1 and HSP70-1 gene overexpression *in vitro* clearly showed that HSP70-1 was essential for the IL-5-induced angiogenic responses through eNOS phosphorylation at Ser-1177 in HUVECs. However, AKT signaling was not involved in the HSP70-1-mediated angiogenic function of IL-5. The results from aortic ring assay and a plug *in vivo* experiment in HSP70-1-deficient mice and HSP70-1 overexpression transgenic mice also demonstrated that HSP70-1 is a necessary and sufficient regulator for IL-5-induced angiogenic responses. However, it was interesting that the lack of HSP70-1 had no effect on either the *in vitro* or *in vivo* angiogenic responses induced by VEGF, suggesting that the IL-5-mediated angiogenic effect is likely, at least in part, caused by a direct expression of the HSP70-1 gene.

Our data demonstrated the involvement of HSP70-1 and eNOS in IL-5-induced angiogenesis. However, overexpression of the HSP70-1 gene did not recover the diminished proliferation, migration, invasion, or tube formation of HUVECs induced by IL-5 in the presence of IL-5Rα silencing, suggesting that HSP70-1 alone would not be sufficient to affect the proliferation, migration, invasion or tube formation of HUVECs. Previous studies have shown that endothelium-derived NO plays a critical role in both angiogenesis and vascular remodeling^20^,^37^. The results from the present study indicated that proliferation and tube formation in HUVECs was not stimulated by eNOS gene overexpression. In addition, we found that eNOS gene overexpression induced the migration and invasion of HUVECs. Treatment with IL-5 did not further stimulate the proliferation and tube formation of HUVECs overexpressing the eNOS gene. In addition, enforced overexpression of the eNOS gene could not rescue the decreased proliferation, migration, invasion, and tube formation of HUVECs by IL-5 in the presence of HSP70-1 silencing. These data demonstrate that the eNOS gene alone would not be sufficient to change the proliferation, migration, invasion, and tube formation in IL-5-treated HUVECs.

Our data showed that IL-5 stimulated angiogenic responses via eNOS expression, suggesting that inflammatory cytokine may be a critical risk factor for tumor-associated angiogenesis. In addition, our *in vitro* studies indicated that HSP70-1 is deeply associated with angiogenic responses induced by IL-5. We, therefore, investigated the effect of genetic model of HSP70-1 on IL-5-induced angiogenic responses. We directly exhibited that HSP70-1 transgenic mice significantly enhanced angiogenic function of IL-5 compared with those of WT mice. Furthermore, our results showed that eNOS is a physiological binding partner of HSP70-1 protein in IL-5-induced angiogenic responses of HUVECs. Based on these results, we predict that up-regulation of HSP70-1 might stimulate the eNOS signaling in response to IL-5, resulting in the enhanced association of eNOS/HSP70-1 and subsequent activation of angiogenic responses. Additional study will be needed to elucidate the detailed mechanism of HSP70-1 expression in the IL-5-stimulated angiogenic responses.

We here investigated how signaling pathways resulted in the induction of HSP70-1 in IL-5-treated HUVECs. Previous study have identified transcription factor AP-1 that is involved in the regulation of HSP70 expression by cadmium in HepG2 cells^38^. It is known that ERK and AKT can induce DNA binding activity of AP-1 through direct phosphorylation of c-Jun^39^,^40^. We found that AP-1 is essential for the IL-5-induced HSP70-1 expression through ERK and AKT signaling in HUVECs. These results indicate that AP-1 may up-regulate the HSP70-1 expression induced by ERK and AKT signaling in IL-5-treated HUVECs. These data support a possible role for AP-1 in the IL-5-related angiogenesis mediating the expression of HSP70-1.

There is much evidence that validates VEGF as a mediator of angiogenesis^1^,^2^,^22^. Subsequently, anti-cancer agents that inhibit the signaling pathways of VEGF and its receptors have been developed^1^,^2^,^22^. Similar to other agents, drug resistance to anti-angiogenic molecules occurred during treatment, and resulted in the progression of disease^22^. Therefore, there is an urgent need to clarify the detailed mechanisms that regulate the VEGF-independent angiogenic signaling pathways. Cumulative studies have provided the prominent role of the cytokines that are secreted by inflammatory cells during angiogenesis^2^,^20^,^21^. Although several types of VEGF-independent angiogenic factors, such as FGF, IL-32, and IL-33, have been identified^2^,^20^,^21^, the exact molecular mechanism in mediating VEGF-independent angiogenesis remains to be elucidated. The data from the present study now provides novel evidence that IL-5 induces VEGF-independent angiogenic effects via the expression of HSP70-1. It is possible that this protein might play a role in potential therapies targeting anti-angiogenesis. It will be interesting to investigate whether HSP70-1 is involved in other forms of the angiogenic factor-induced control of angiogenesis.

These results suggest that HSP70-1 is required for IL-5-induced angiogenesis through eNOS pathways, and the present study is the first direct evidence between molecular chaperones and VEGF-independent angiogenic function by inflammatory cytokines, which might provide insight into the formation of neo-vasculature during the progression of inflammatory-mediated vascular diseases.

## Materials and Methods

### Cell culture

Primary HUVECs were obtained from Lonza (Walkersville, MD, USA). Cells were maintained on plates coated with 0.1% gelatin (Sigma, San Diego, CA, USA) in endothelial basic medium (EBM) and were maintained in EGM^TM^-2 Bulletkit^TM^ (Lonza) at 37 °C in a 5% CO_2_ humidified incubator. All experiments were carried out between passages 2 to 5. The cells have been used finished the mycoplasma contamination test.

### Mice

All experimental procedures were performed in accordance with guidelines set by the Animal Care and Use Committee of Chungbuk National University. We obtained the HSP70-1 knockout mice used in this study from Dr. Jeong-Sun Seo, at Seoul National University. The method used to generate the HSP70-1 knockout mice used in this study has been previously described^14^,^15^. IL-5-deficient (IL5tm1Kopf/J) mice with a C57BL/6 background and HSP70-1-transgenic (Tg-(Hspa1a-luc,-EGFP)2Chco/J) mice with a FVB/NJ background were purchased from The Jackson Laboratory. Mice were housed at 23 ± 5 °C under a 12 h dark/light cycle. All mice were provided with water and standard chow ad libitum for 1 week before experiments. Mice were anesthetized with ketamine (100 mg/kg, Yuhan Corporation, Seoul, Republic of Korea), and xylazine (10 mg/kg, Bayer Korea Ltd., Seoul, Republic of Korea), intraperitoneal (i.p.) before surgery. Body temperature was maintained at 37 ± 0.5 °C throughout surgery using a thermostatically controlled warming plate. For the plasma collection, blood was obtained from retroorbital plexus using microhematocrit capillary tubes coated with heparin. Blood was centrifuged at 3,000 rpm for 10 min at 4 °C. Subsequently, plasma was stored at −80 °C for NO determination.

### Reverse transcriptase–polymerase chain reaction (RT-PCR) analysis

Total RNA was isolated from treated cells using the TRIzol reagent (Life Technologies, NY) according to the manufacturer’s protocol. The synthesized cDNA was amplified using specific primers for HSP70-1, VEGF-A, bFGF, Ang-1, Ang-2, and actin. The primers used had the following sequences: HSP70-1 (447 bp), 5′-TTT CGA GAG TGA CTC CCG TT-3′ (sense) and 5′-AAG GCC AGT GCT TCA TGT C-3′ (antisense); VEGFA (233 bp), 5′-TGTCTATCAGCGCAGCTACTGCCAT-3′ (sense) and 5′-GGAAGCTCATCTCTCCTATGTGCTG (antisense); b-FGF (270 bp), 5′-AGAGCGACCCTCACATCAAGCTAC-3′ (sense) and 5′-CTTTCTGCCCAGGTCCTGTTTTGGA-3′ (antisense); Ang-1 (404 bp), 5′-TATGCCAGAACCCAAAAAGG-3′ (sense) and 5′-GGGCACATTTGCACATACAG-3′ (antisense); Ang-2 (426 bp) 5′-TGGGATTTGGTAACCCTTCA-3′ (sense) and 5′-CCTTGAGCG AATAGCCTGAG-3′ (antisense); and actin (235 bp), 5′-CCC AGA TCA TGT TTG AGA CCT-3′ (sense) and 5′-ATG TCA CGC ACG ATT TCC C-3′ (antisense). The PCR conditions were optimized to: HSP70-1, 35 cycles of denaturing at 95 °C for 20 s, annealing at 55 °C for 30 s, and extension at 72 °C for 40 s; and, actin, 25 cycles of denaturing at 95 °C for 30 s, annealing at 57 °C for 30 s, and extension at 72 °C for 20 s. The quality and integrity of The RNA were confirmed by 1% agarose gel electrophoresis and ethidium bromide staining, followed by visual examination under ultraviolet light.

### Immunoblot

Cells were treated with IL-5 in EBM containing 0.1% FBS and then lysed with the cell lysis buffer (containing, in mmol/L, HEPES [pH 7.5] 50, NaCl 150, EDTA 1, EGTA 2.5, DTT 1, β-glycerophosphate 10, NaF 1, Na_3_VO_4_ 0.1, and phenylmethylsulfonyl fluoride 0.1, 10% glycerol, 0.1% Tween-20, 10 μg/mL of leupeptin, and 2 μg/mL of aprotinin). The lysates were centrifuged at 12,000 rpm for 20 min at 4 °C. The protein concentration of the supernatant was measured with a BCA Protein Assay Reagent (Pierce, Rockford, IL, U.S.). Equal amounts of cellular proteins (30 μg) were separated by 6~15% SDS-PAGE and transferred to nitrocellulose membranes. After blocking for 1 h in 5% skim milk, the membranes were incubated with primary antibodies against phospho-eNOS (S1177), eNOS, phospho-Akt (T473), Akt, phospho-ERK, ERK, Hsp70, IL-5, IL-5Rα, VEGF-A, VEGF-C, bFGF, Ang-1, Ang-2, and actin, overnight at 4 °C. The membranes were then incubated with peroxidase-conjugated secondary antibodies for 1 h at room temperature. The immunocomplexes were detected using ECL Plus Western Blotting Detection Reagents (Amersham Biosciences, Piscataway, NJ, U.S.). Bands were quantified by scanning densitometry and analyzed using Image Quant software.

### Small-interfering RNA Transfection

Cells were transfected with specific or non-specific controlled small interfering RNA (siRNA; Genolution, Seoul, South Korea) at concentrations ranging from 10 to 30 nM using G-fectin transfection reagent (Genolution), according to the manufacturer’s protocols^8^. After incubation for 24 h, gene expression was analyzed by RT-PCR or immunoblot. The siRNA that was sequences used had the following sequences: HSP70-1 (#1), 5′ GUUUGUCAGUUCUCAAUUUUU 3′ (Sense) and 5′ AAAUUGAGAACUGACAAACUU 3′ (antisense), HSP70-1 (#2), 5′ CCAUCUUACGACUAUUUCUUU 3′ (Sense) and 5′ AGAAAUAGUCGUAAGAUGGUU 3′ (antisense), scramble (scrambled siRNA), 5′ CCUCGUGCCGUUCCAUCAGGUAGUU 3′ (Sense) and 5′ CUACCUGAUGGAACGGCACGAGGUU 3′ (antisense), IL-5Rα (#1), 5′ CCUGUCAAUUUCACCAUUAUU 3′ (Sense) and 5′ UAAUGGUGAAAUUGACAGGUU 3′ (antisense). IL-5Rα (#2), 5′ GAAUGUUAAUCUAGAAUAUUU 3′ (Sense) and 5′ AUAUUCUAGAUUAACAUUCUU 3′ (antisense). After the indicated incubation with IL-5 for 24 h, the cells were analyzed for immunoblot, proliferation, tube formation, invasion, and wound-healing migration.

### [^3^H]thymidine incorporation

Cells were cultured on the surface of the 0.1%-coated gelatin at 80 to 90% confluence, then starved in culture in EBM medium with 0.1% FBS for 6 h. Cells were incubated with various concentrations of IL-5 for 24 h. [^3^H]thymidine incorporation assay was performed to determine the cell proliferation of IL-5 on HUVEC^41^.

### Invasion assay

Cells incubated in EBM containing 1% FBS for 3 hours were trypsinized and resuspended with IL-5 in the upper portion of the 8 μm pore size transwell inserts with EBM containing 0.1% FBS for 12 h. Cells had to pass through a polycarbonate membrane and a thin layer of an ECM Matrix-like material. Inserts were fixed with 4% PFA and stained with 0.1% crystalviolet. The photographs of cells that attached to the lower side of the inserts were taken through an inverted microscope (x100 magnification) and analyzed using Image Quant software.

### Cell migration assay

Cells were placed on 6-well plates and grown to 90%. After the cells were incubated in EBM containing 1% FBS for 3 hours, they were scratched using a 200 μl pipette tip and were then incubated with IL-5 in EBM containing 0.1% FBS for 24 h. Cell migration was observed via inverted microscope (x40 magnification), and was calculated using the following formula:


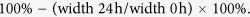


### Colony tube formation assay

Cells incubated in EBM containing 1% FBS for 3 hours were trypsinized and seeded onto BD matrigel matrix growth factor reduced-coated 24-well plates, and treated with IL-5 in EBM containing 0.1% FBS. After incubation for 8 h, tube formation was observed using an inverted microscope (x40 magnification) and was quantified by measuring the tube length using Image-Pro Plus software.

### Immunoprecipitation

Cells were prepared with lysis buffer. Cell lysates then were clarified by centrifugation at 12,000 rpm for 20 min at 4 °C. The supernatants were incubated with anti-HSP70 antibody (1:100) overnight at 4 °C. Immunocomplexes were mixed with ProteinG Plus/Protein A agarose for 4 h at 4 °C and then washed 3 times with 1X PBS. The final pellet was resuspended in 20 μL of lysis buffer with Laemmli sample buffer and heated for 10 min at 100 °C.

### ELISA immunoassay for IL-5

HUVECs were cultured with endothelial basic medium (EBM) and EGM^TM^-2 Bulletkit^TM^ (Lonza). Aliquots of the cell culture supernatant were separated and subjected to assay for the levels of IL-5 using a Human IL-5 Immunoassay kit (R&D Systems, Minneapolis, MN). Supernates from human peripheral blood mononuclear cells (PBL) were supplied by the kit, and used as a positive control.

### Plasmids and transfections

Plasmid encoding the human eNOS (NOS3) in pTZ18RP1 vector and HSP70-1 (HSPA1A) plasmid in pOTB7 vector were provided from Korea Human Gene Bank, Medical Genomics Research center, KRIBB, Korea. The transfections were performed using Lipofectamine^®^3000 reagent according to the manufacturer’s protocols. Plasmids were transfected in 70–90% confluent HUVEC with complexes of 2 μL lipofectamine^®^3000 reagent in Opti-MEM^®^ medium. After transfection for 24 to 48 h, the expressions of eNOS and HSP70-1 were determined via immunoblot.

### Aortic ring assay

Aortas isolated from 2-month-old C57BL/6 mice were sectioned into 1–1.5 mm long rings, and then placed on matrigel pre-coated wells. Each well containing the aortic rings was incubated in medium containing IL-5, VEGF (50 ng/mL) or IL-5 antibody (2 μg/ml). After 9 days, a microscope was used to quantify the total length of microvessel sprouting via Image-Pro Plus software (Media Cybernetics, USA). Three independent experiments were performed. All animal experiments were performed with the approval of the Animal Care and Use Committee of Chungbuk National University.

### *In vivo* matrigel plug assay

2-month-old C57BL/6 mice were injected subcutaneously with 0.5 ml of Matrigel containing heparin (10 U/ml), IL-5, VEGF (50 ng/mL) or IL-5 antibody (2 μg/ml). After 7 days, the matrigel plugs were removed and photographed. The quantification of vascularization was determined by measuring the hemoglobin content using the Drabkin method (Drabkin reagent kit 525, Sigma-Aldrich, Louis, MO). The infiltrating endothelial cells were identified by immunochemistry using a CD-31 antibody. Confocal microscopy was performed using previously described antibody-conjugated QD565 nanoparticles^7^,^8^.

### Statistical analysis

The results were recorded as the mean ± SE. Data were analyzed using factorial ANOVA analysis and a Fisher’s least significant difference test. Differences with *P* < 0.05 were considered statistically significant.

## Additional Information

**How to cite this article:** Park, S. L. *et al*. HSP70-1 is required for interleukin-5-induced angiogenic responses through eNOS pathway. *Sci. Rep.* **7**, 44687; doi: 10.1038/srep44687 (2017).

**Publisher's note:** Springer Nature remains neutral with regard to jurisdictional claims in published maps and institutional affiliations.

## Supplementary Material

Supplementary Information
