# Supplementary material for: HSP70-1 is required for interleukin-5-induced angiogenic responses through eNOS pathway
Source: Sci Rep. 2017 Mar 20;7:44687. doi: 10.1038/srep44687 (PMC5357797; doi:10.1038/srep44687)
Supplement: Supplementary Information [file srep44687-s1.doc]

**Supplementary information**

**HSP70-1 is required for interleukin-5-induced angiogenic responses through eNOS pathway**

**Running title: IL-5 and HSP70-1 in angiogenesis**

Sung Lyea Park^1*^, **Tae-Wook Chung^2^**^*^, Sangtae Kim^3^, Byungdoo Hwang^1^, Jung Min Kim^4^, **Hwan Myung Lee**^5^, Hee-Jae Cha^6^, Yoonhee Seo^7^, Soo Young Choe^7,8^, Ki-Tae Ha**^2^**, Gonhyung Kim^9^, Seok-Joong Yun^10^, Sung-Soo Park^11^, Yung Hyun Choi^12^, Bo Kyung Kim^13^, Won-Tae Kim^10^, Eun-Jong Cha^14^, Cam Patterson^15^, Wun-Jae Kim^10¶^ , and Sung-Kwon Moon^1¶^

^1^ Department of Food and Nutrition, Chung-Ang University, Anseong 456-756, Korea; ^2^ School of Korean Medicine and Healthy Aging Korean Medicine Research Center, Pusan National University, Yangsan, 626-780, Republic of Korea; ^3^ Biomedical Research Institute, Bundang Hospital, Seoul National University, Korea 647-707; ^4^ NAR Center, Inc., Daejeon Oriental Hospital of Daejeon University, Daejeon 301-724, Republic of Korea; ^5^ **Department of Cosmetic Science, Hoseo University,** Asan-si 31499, Republic of Korea; ^6^ Department of Parasitology and Genetics, Kosin University College of Medicine, Busan 602-702, Republic of Korea; ^7^ EBO Co., Ltd., Future Convergence Technology Center, Cheongwon-Gun, Chungbuk, Korea; ^8^ Department of Biology, Chungbuk National University, Cheongju, Chungbuk, Korea; ^9^ Department of Veterinary Surgery, College of Veterinary Medicine, Chungbuk National University, Cheongju, Chungbuk, Korea; ^10^ Personalized Tumor Engineering Research Center, Department of Urology, Chungbuk National University, Cheongju, Chungbuk 361-763, South Korea; ^11^ Department of Food Science and Nutrition, Jeju National University, Jeju, Jeju Special Self-Governing Province, 690-756, South Korea; ^12^ Department of Biochemistry, College of Oriental Medicine, Dongeui University, Busan 614-052, South Korea; ^13^ Department of Physiology, College of Medicine, Konkuk University, Seoul 143-701, Republic of Korea; Catholic University of Daegu School of Medicine, Daegu 705-718, Republic of Korea; ^14^ Department of Biomedical Engineering, Chungbuk National University, Cheongju 361-763, Korea; ^15^ NewYork-Presbyterian Hospital, New York, NY 10065, USA

**Supplementary Methods**

**Materials**

Recombinant human IL-5 and IL-5 antibodies were purchased from R&D Systems (Minneapolis, MN). Polyclonal antibodies to phospho-eNOS (S1177), eNOS, phospho-AKT, AKT, phospho-ERK, and ERK were obtained from Cell Signaling (Danvers, MA). U0126, wortmannin, L-NAME, and ProteinG Plus/Protein A agarose suspensions were obtained from Calbiochem (San Diego, CA). Hsp70, IL-5, IL-5Rα, VEGF-A, VEGF-C, bFGF, Ang-1, Ang-2, and peroxidase-conjugated secondary antibodies were purchased from Santa Cruz Biotechnology (Santa Cruz, CA). The actin antibody was obtained from Sigma (St. Louis, Mo). Lipofectamine®3000 reagent were purchased from Invitrogen Life Technologies (Italy).

**Nuclear extracts and electrophoretic mobility shift assay (EMSA)**

Cells were harvested, washed and resuspended in a buffer containing 10 mM HEPES (pH 7.9), 10 mM KCl, 1 mM DTT, 0.5 mM PMSF, 0.1 mM EDTA, and 0.1 mM EGTA. After incubation on ice for 15 min, cells were vigorously mixed in the presence of 0.5% Nonidet NP-40. Then, the nuclear pellet was centrifuged, and extracted in a buffer containing 20 mM HEPES (pH 7.9), 400 mM NaCl, 1 mM DTT, 1 mM PMSF, 1 mM EDTA, and 1 mM EGTA at 4 °C for 15 min. The nuclear protein concentrations were determined by protein assay. EMSA probes consist of annealed oligonucleotides of the either an AP-1 consensus binding sequence 5'-CGCTTGATGAGTCAGCCGGAA-3' (Promega), or a Sp-1 consensus binding sequence 5'-ATTCGATCGGGGCGGGGCGAGC-3' (Promega), which was labeled with γ-32^P^-ATP (Perkin Elmer Life and Analytical Sciences, Waltham, MA) using T4 polynucleotide kinase (Promega, Madison, WI). Then, the nuclear extract (10–20 μg) was incubated with labeled probes at 4 °C for 20 min in a buffer (25 mM HEPES buffer (pH 7.9), 0.5 mM EDTA, 0.5 mM DTT, 50 mM NaCl, and 2.5% glycerol) with 2 μg of poly dI/dC. The binding products were resolved by electrophoresis at 4 °C on a 6% polyacrylamide gel. The gel was dried and exposed to X-ray film overnight. The specificity of both AP-1 and Sp-1 binding activity was determined with the 100-fold excess of either unlabeled AP-1 or Sp-1 oligonucleotide respectively as competitor.

**RNA preparation for microarray analysis**

Total RNA was extracted using Trizol (Invitrogen Life Technologies, Carlsbad, USA), then it was purified using RNeasy columns (Qiagen, Valencia, USA) according to the manufacturers’ protocol. After processing with DNase digestion and executing cleanup procedures, the RNA samples were quantified by aliquot and stored at -80 °C until use. For quality control, RNA purity and integrity were evaluated by denaturing gel electrophoresis, OD 260/280 ratio, and analyzed using an Agilent 2100 Bioanalyzer (Agilent Technologies, Palo Alto, USA).

**Labeling and purification for microarray analysis**

Total RNA was amplified and purified using an Ambion Illumina RNA amplification kit (Ambion, Austin, USA) to yield biotinylated cRNA according to the manufacturer’s instructions. Briefly, 550 ng of total RNA was reverse-transcribed to cDNA using a T7 oligo(dT) primer. Second-strand cDNA was synthesized, *in vitro* transcribed, and labeled with biotin-NTP. After purification, the cRNA was quantified using an ND-1000 Spectrophotometer (NanoDrop, Wilmington, USA).

**Hybridization and data export for microarray analysis**

750 ng of labeled cRNA samples were hybridized to each Human HT12 expression v.4 bead array for 16-18 h at 58 °C, according to the manufacturer's instructions (Illumina, Inc., San Diego, USA). Detection of array signals was carried out using Amersham fluorolink streptavidin-Cy3 (GE Healthcare Bio-Sciences, Little Chalfont, UK) following the bead array manual. Arrays were scanned with an Illumina bead array reader confocal scanner according to the manufacturer's instructions

**Raw data preparation and statistical analysis (example) for microarray datasets**

The quality of hybridization and overall chip performance was monitored by visual inspection of both internal quality control checks and the raw scanned data. Raw data were extracted using the software provided by the manufacturer (Illumina GenomeStudio v2011.1 (Gene Expression Module v1.9.0). The signal values of probes were transformed via logarithm and normalized via the quantile method. Statistical significance of the expression data was determined using the Fold change. Gene-Enrichment and Functional Annotation analysis for a significant probe list was performed using DAVID (http://david.abcc.ncifcrf.gov/). All data analysis and visualization of differentially expressed genes were conducted using R 2.15.0 ([www.r-project.org](http://www.r-project.org/)).

**Nitric oxide (NO) production**

NO production was used by modified griess reagent method (Abcam, Cambridge, USA). Nitrite content in cell culture supernatant or plasma was measured to indi­rectly reflect the content of NO. The 50 μl of sample (culture supernatant or plasma) was added to an equal volume of Griess reagent. After 10 min of incubation at room temperature, an automatic microplate reader was utilized to detect absorbance at 540 nm. NO levels were determined by a calibrated curve using sodium nitrite standards.

**Intracellular NO detection**

HUVECs were treated with IL-5 for 4 h, and followed by incubation of 5 μM of 4-amino-5-methylamino-2’,7’-difluorofluorescein (DAF-FM) diacetate (Molecular Probes Inc) for 1 h at 37 °C. After removal of the excess probe, the fluorescence images were photographed from randomly selected cells using a Nikon exlipse Ti fluorescence microscope (100 X). The intensity levels of intracellular NO were quantified from the DAF-FM fluorescence.

**Supplementary Figures**

**Supplementary Figure S1**


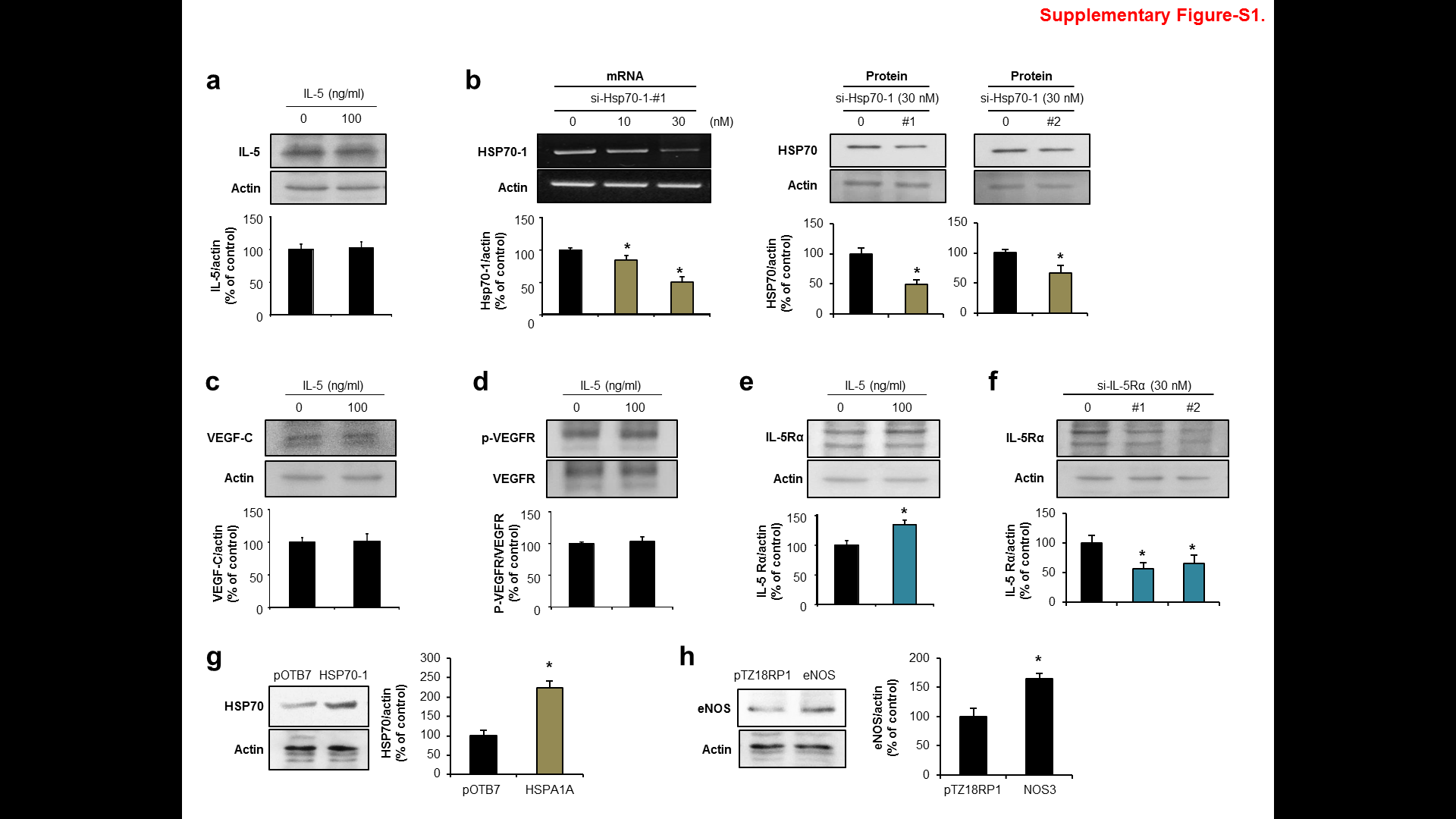


**Figure S1. Expression of IL-5, HSP70-1, VEGF-C and VEGFR phosphorylation in IL-5-treated HUVECs.** (**a**) After starvation, HUVECs were treated with IL-5 for 24 h. Immunoblot was performed with specific antibody for IL-5. (**b**) After transfection of HSP70-1 siRNA, expression levels of HSP70-1 were confirmed by RT-PCR and immunoblot. (**c,d**) After starvation, HUVECs were stimulated with IL-5 for 24 h. Immunoblot was performed using antibodies specific for VEGF-C, phospho-VEGFR and VEGFR. (**e**) Expression of IL-5Rα was performed in IL-5-treated HUVECs using immunoblot analysis. (**f**) HUVECs were transfected with IL-5Rα siRNA. At 24 h after transfection, protein levels of IL-5Rα were analyzed by immunoblot. (**g,h**) After transfection of either the HSP70-1 gene or eNOS genes, expression levels of HSP70-1 and eNOS were confirmed by immunoblot. All data are reported as the means ± SE from three independent experiments. **P* < 0.05 compared with control.

**Supplementary Figure S2**


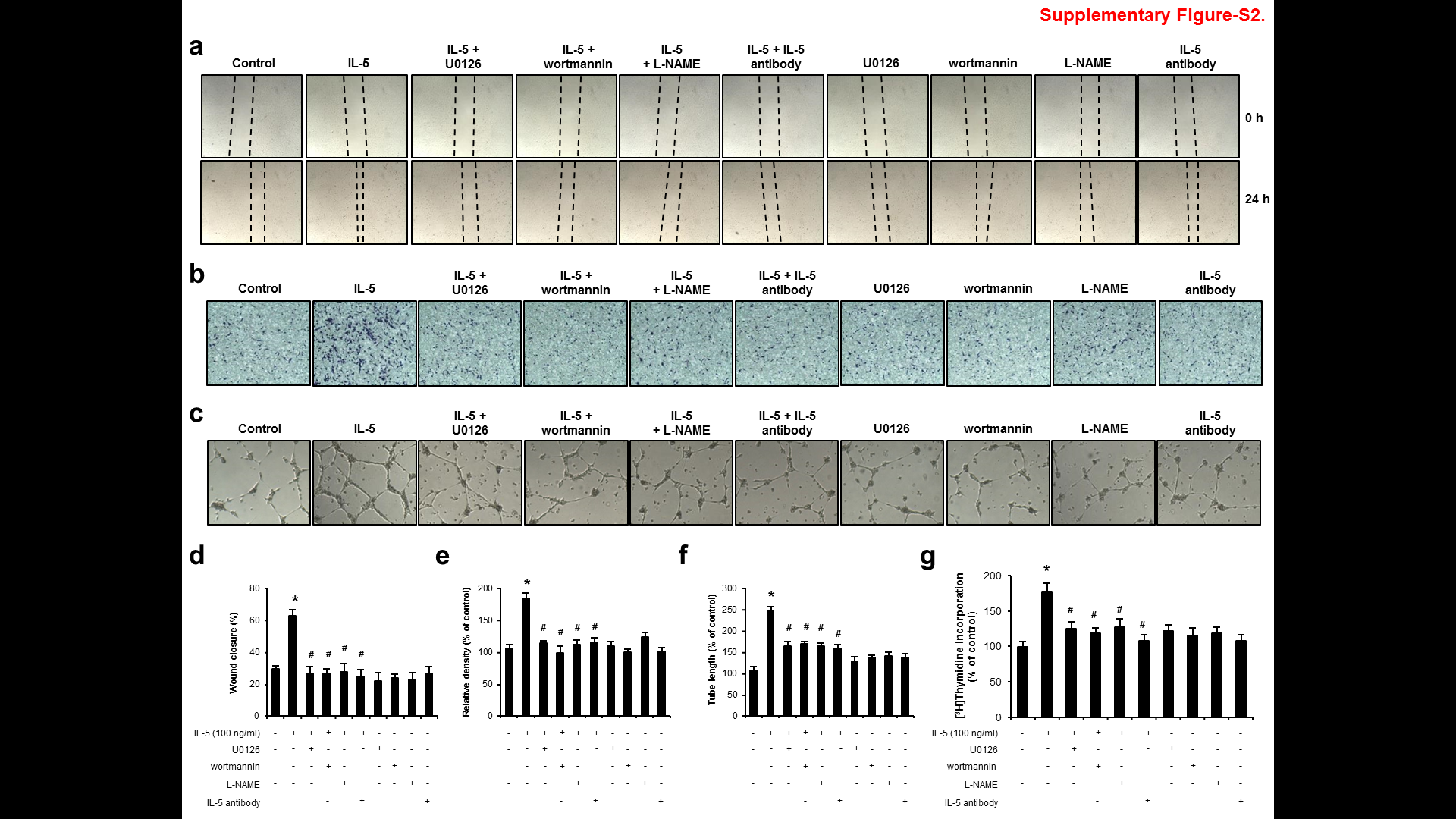


**Figure S2. Effect of U0126, wortmannin, L-NAME, and IL-5 antibody in IL-5-induced angiogenic responses.** HUVECs were pre-incubated in serum free medium with U0126 (10 μM), wortmannin (10 μM), L-NAME (100 μM), and IL-5 antibody (1 μg/ml) for 40 min followed by addition of IL-5 (100 ng/ml) and incubation for additional 24 h. (A-D) The proliferation, migration, invasion, and colony tube formation assay was performed, as described in Materials and Methods. All data are reported as the means ± SE from three independent experiments. **P* < 0.05 compared with control, ^#^*P* < 0.05 compared with IL-5 treatment.

**Supplementary Figure S3**


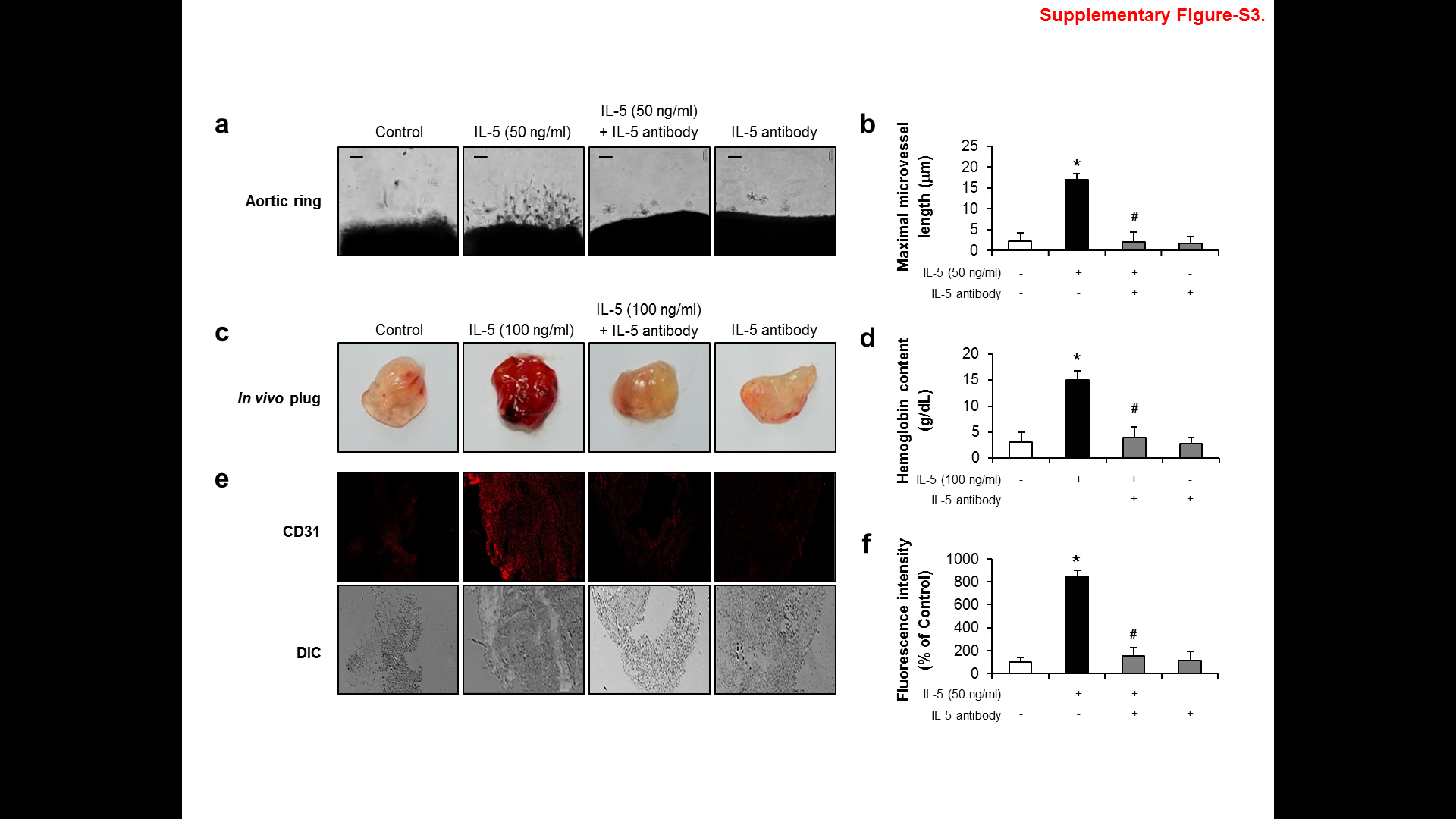


**Figure S3. Effect of IL-5 neutralizing antibody in IL-5-induced microvessel formation of angiogenesis *ex vivo* and *in vivo*.** (**a, b**) Angiogenesis *ex vivo* aortic ring assay and the number of neo-vessel sprouts in an aortic ring assay. (**c, d**) The matrigel plug *in vivo* assay and quantitative analysis of neovessel formation by hemoglobin contents in the matrigel. (**e, f**) Immunostaining of the matrigel plug with CD31 antibody and quantification of the area of CD31-positive vessels. All data are reported as the means ± SE from three independent experiments. **P* < 0.05 compared with control, ^#^*P* < 0.05 compared with IL-5 treatment.

**Supplementary Figure S4**


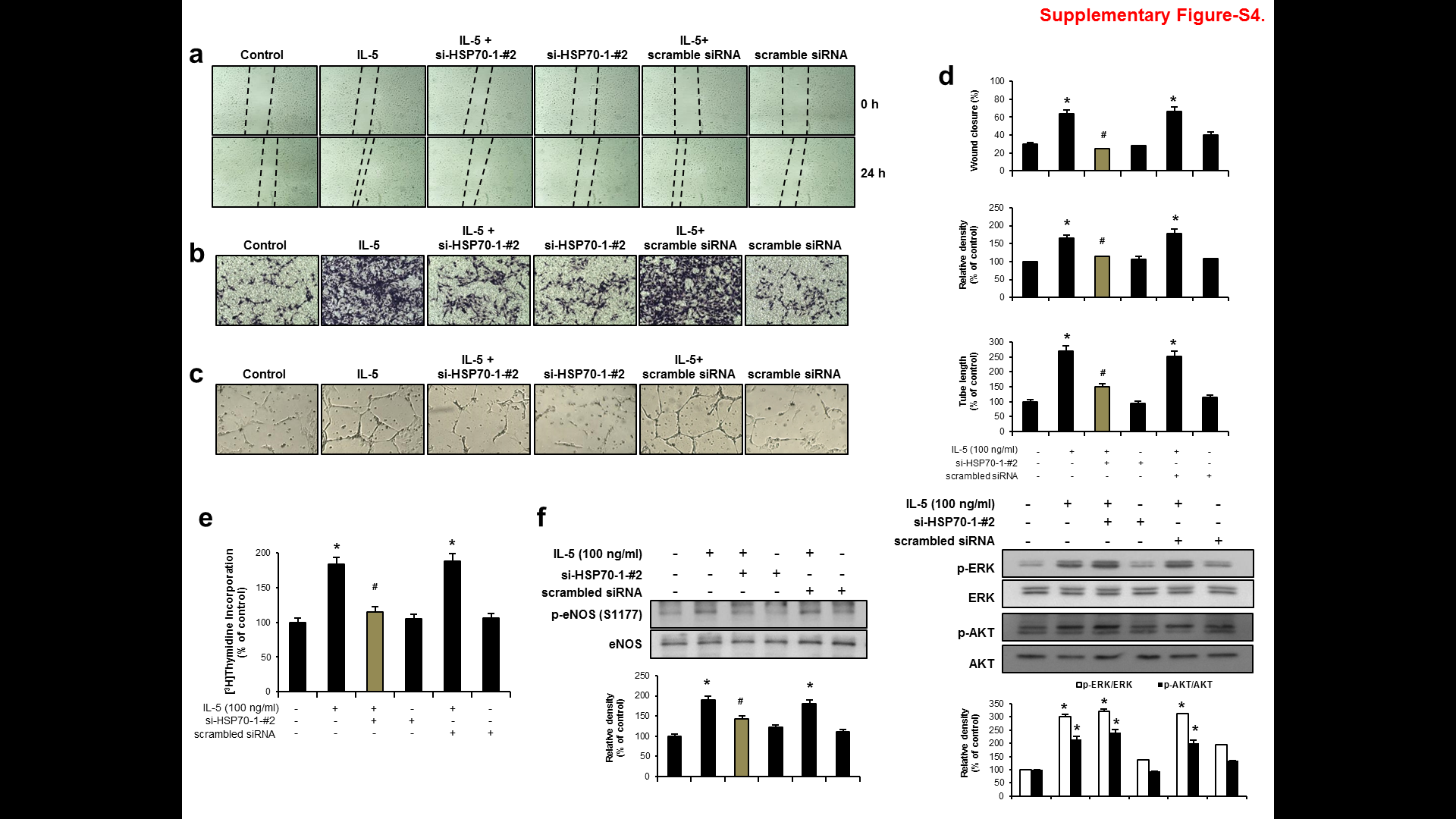


**Figure S4. siRNA-mediated knockdown of HSP70-1 (si-HSP70-1-#2) led to the dysfunction of IL-5-induced proliferation, migration, colony tube formation, and phosphorylation of eNOS in HUVECs.** (**a-e**) IL-5-induced proliferation, migration, invasion, and colony tube formation was determined in HSP70-1 siRNA (si-HSP70-1-#2) transfected HUVECs. (**f**) After transfection of HSP70-1 siRNA (si-HSP70-1-#2) or scrambled siRNA for 24 h, cells were subjected to immunoblot analysis using specific antibodies for ERK1/2, AKT, and eNOS (S1177). All data are reported as the means ± SE from three independent experiments. **P* < 0.05 compared with control, ^#^*P* < 0.05 compared with IL-5 treatment.

**Supplementary Figure S5**


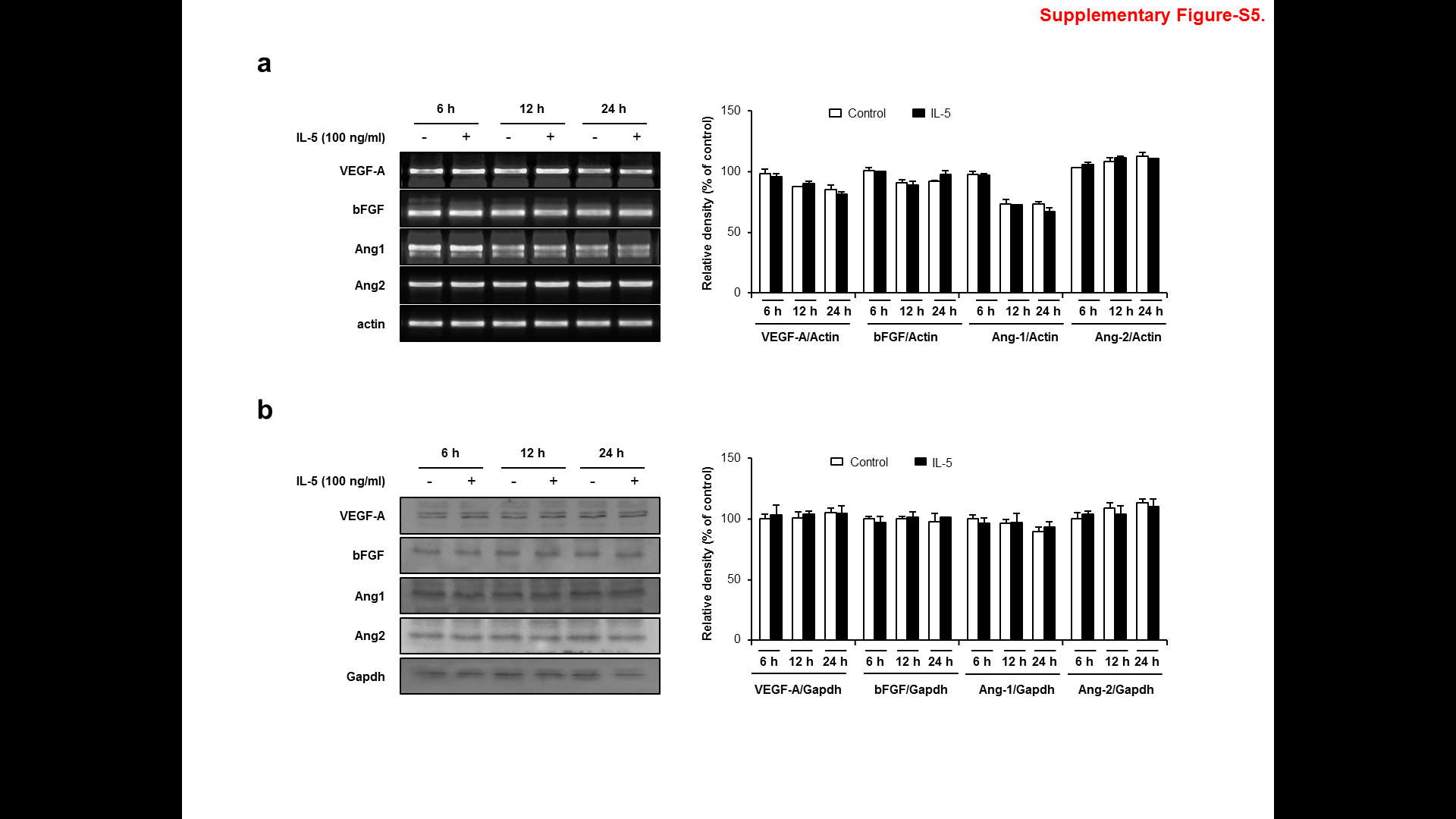


**Figure S5.** **Expression of VEGF-A, bFGF, Ang-1, and Ang-2 in IL-5-treated HUVECs**. (**a,b**) After starvation, HUVECs were stimulated with IL-5 for 24 h. RT-PCR and Immunoblot was performed. All data are reported as the means ± SE from three independent experiments.

**Supplementary Figure S6**


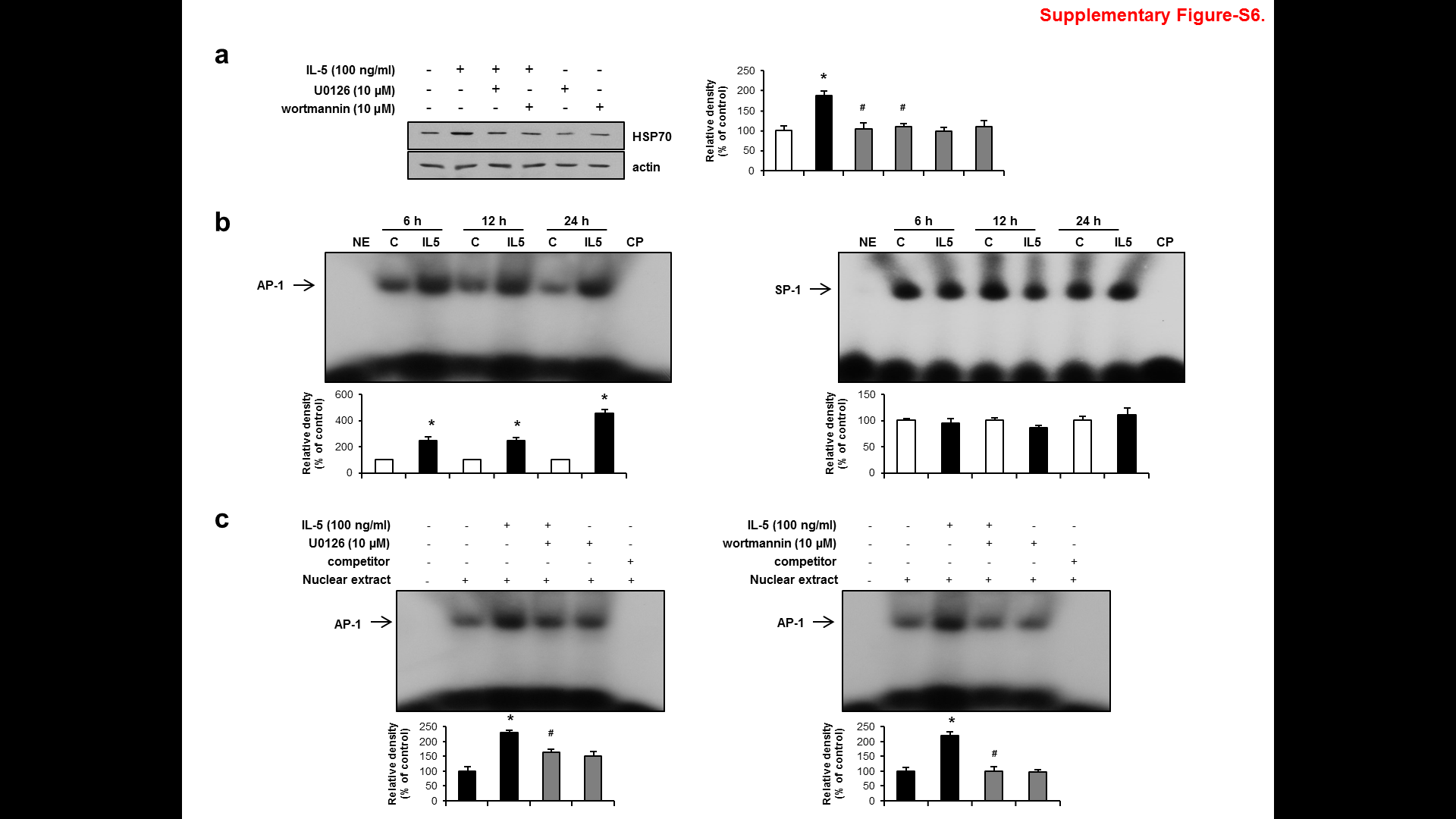


**Figure S6. AP-1 is a main factor in IL-5-induced HSP70-1 expression via ERK1/2 and AKT signaling in HUVECs.** (A) The effect of U0126 (10 μM) and wortmannin (10 μM) in IL-5-induced HSP70-1 expression. (B) After serum-starvation for 24 h, cells were incubated with IL-5 (100 ng/ml) for 24 h, and the binding activity of AP-1 and Sp-1 was determined by EMSA. To stimulate activity of AP-1 and Sp-1, the cells treated with IL-5 were incubated with an unlabeled oligo probes (competitor). (C) Role of U0126 and wortmannin in the induction of AP-1 binding activity in IL-5-treated HUVECs. Cells were cultured with IL-5 (100 ng/ml) in the absence or presence of inhibitors and nuclear proteins were extracted. The AP-1 DNA binding activity was examined by EMSA using radiolabeled oligonucleotide probes and unlabeled oligo probes (competitor). **P* < 0.05 compared with control, ^#^*P* < 0.05 compared with IL-5 treatment.

**Supplementary Figure S7**


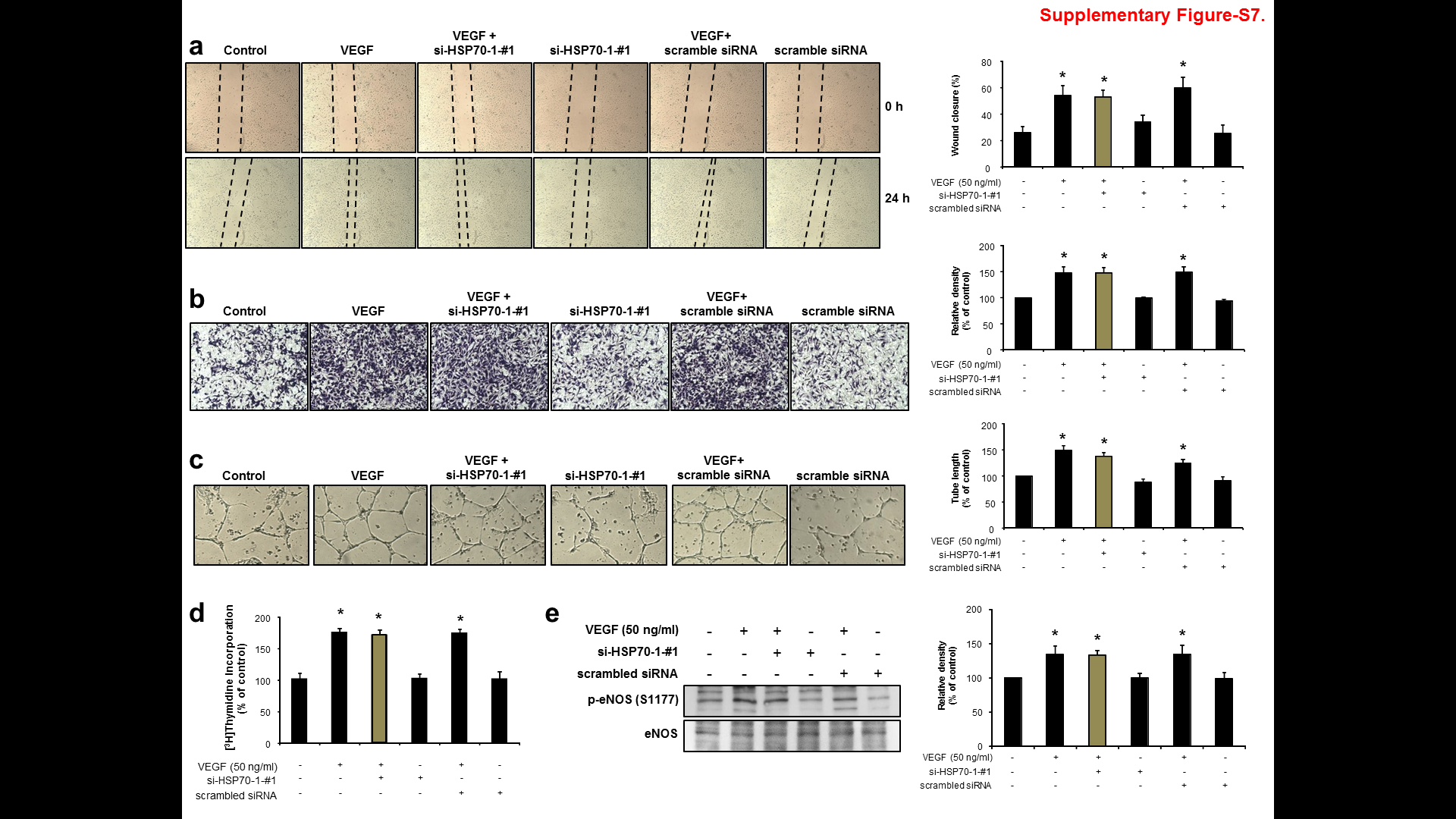


**Figure S7.** **HSP70-1 had no effect on the VEGF-mediated stimulation of angiogenic responses in HUVECs**. (**a-d**) After transfection of either HSP70-1 siRNA (si-HSP70-1-#1) or scrambled siRNA for 24 h, cells were analyzed to evaluate the VEGF-stimulated proliferation, migration, invasion, and colony tube formation. (**e**) Phosphorylation of eNOS induced by VEGF was examined in the either HSP70-1 siRNA (si-HSP70-1-#1) or scrambled siRNA transfected cells. All data are reported as the means ± SE from three independent experiments. **P* < 0.05 compared with control.

**Supplementary Figure S8**


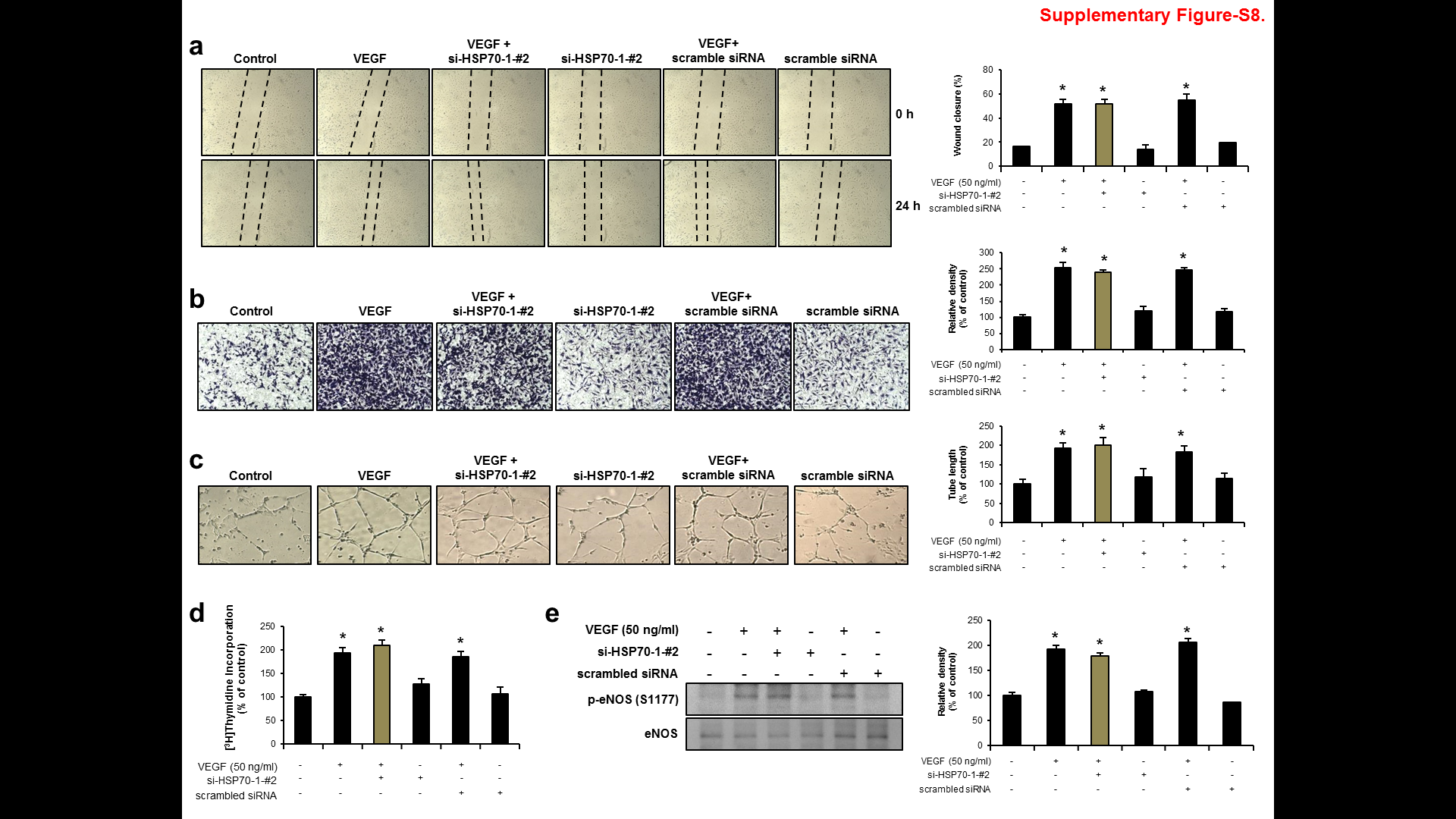


**Figure S8.** **HSP70-1 had no effect on the VEGF-mediated stimulation of angiogenic responses in HUVECs**. (**a-d**) After transfection of either HSP70-1 siRNA (si-HSP70-1-#2) or scrambled siRNA for 24 h, cells were analyzed to evaluate the VEGF-stimulated proliferation, migration, invasion, and colony tube formation. (**e**) Phosphorylation of eNOS induced by VEGF was examined in the either HSP70-1 siRNA (si-HSP70-1-#2) or scrambled siRNA transfected cells. All data are reported as the means ± SE from three independent experiments. **P* < 0.05 compared with control.

**Supplementary Figure S9**


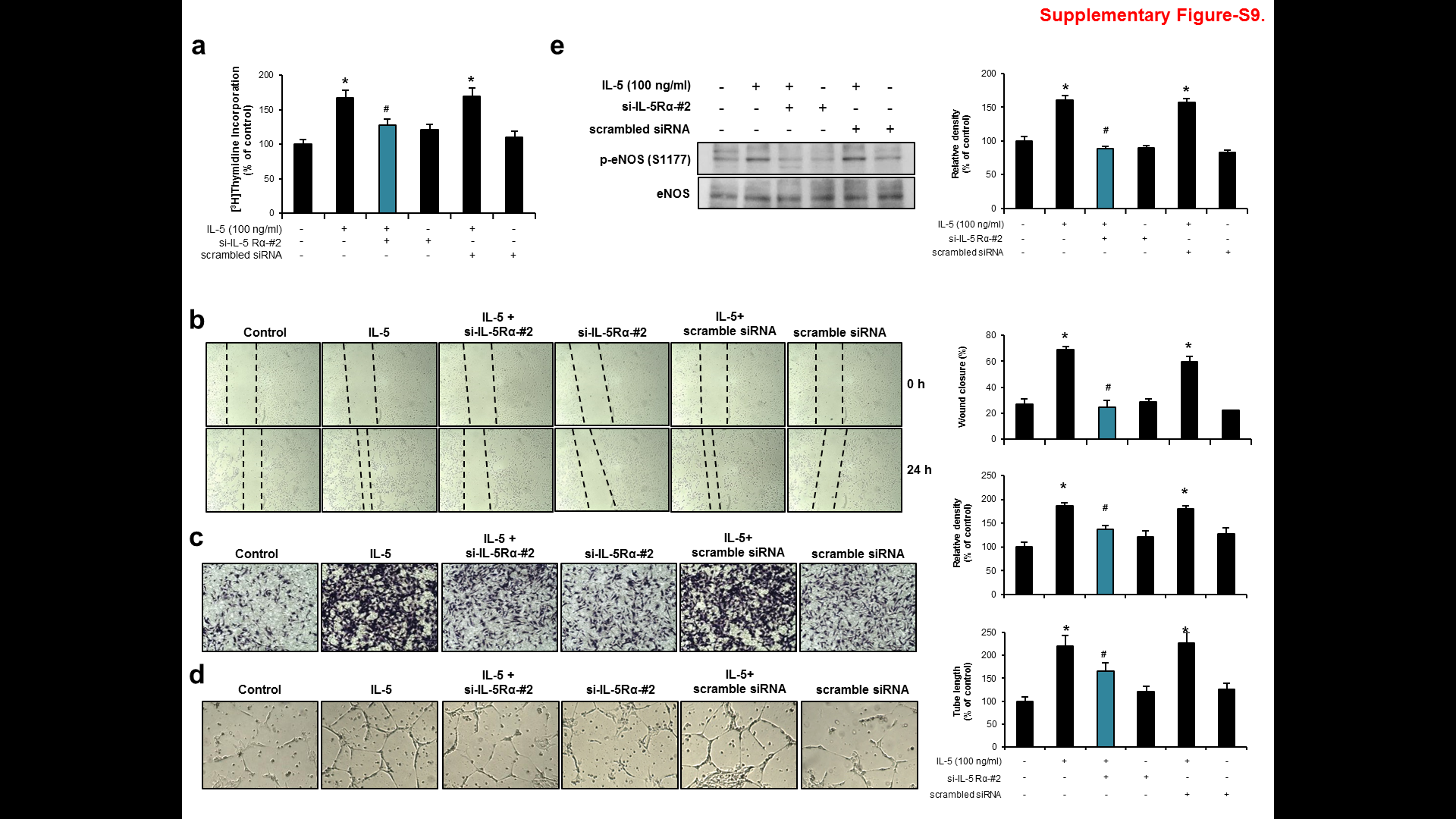


**Figure S9.** **IL-5 induced angiogenic responses via binding of IL-5Rα in HUVECs.** (**a-d**) After transfection of IL-5Rα siRNA (si- IL-5Rα-#2) or scrambled siRNA for 24 h, cells were analyzed to determine the IL-5-stimulated proliferation, migration, invasion, and colony tube formation. (**e**) Immunoblot of phospho-eNOS (S1177) and eNOS in the IL-5Rα siRNA (si- IL-5Rα-#2) or scrambled siRNA transfected cells. All data are reported as the means ± SE from three independent experiments. **P* < 0.05 compared with control, ^#^*P* < 0.05 compared with IL-5 treatment.

**Supplementary Figure S10**


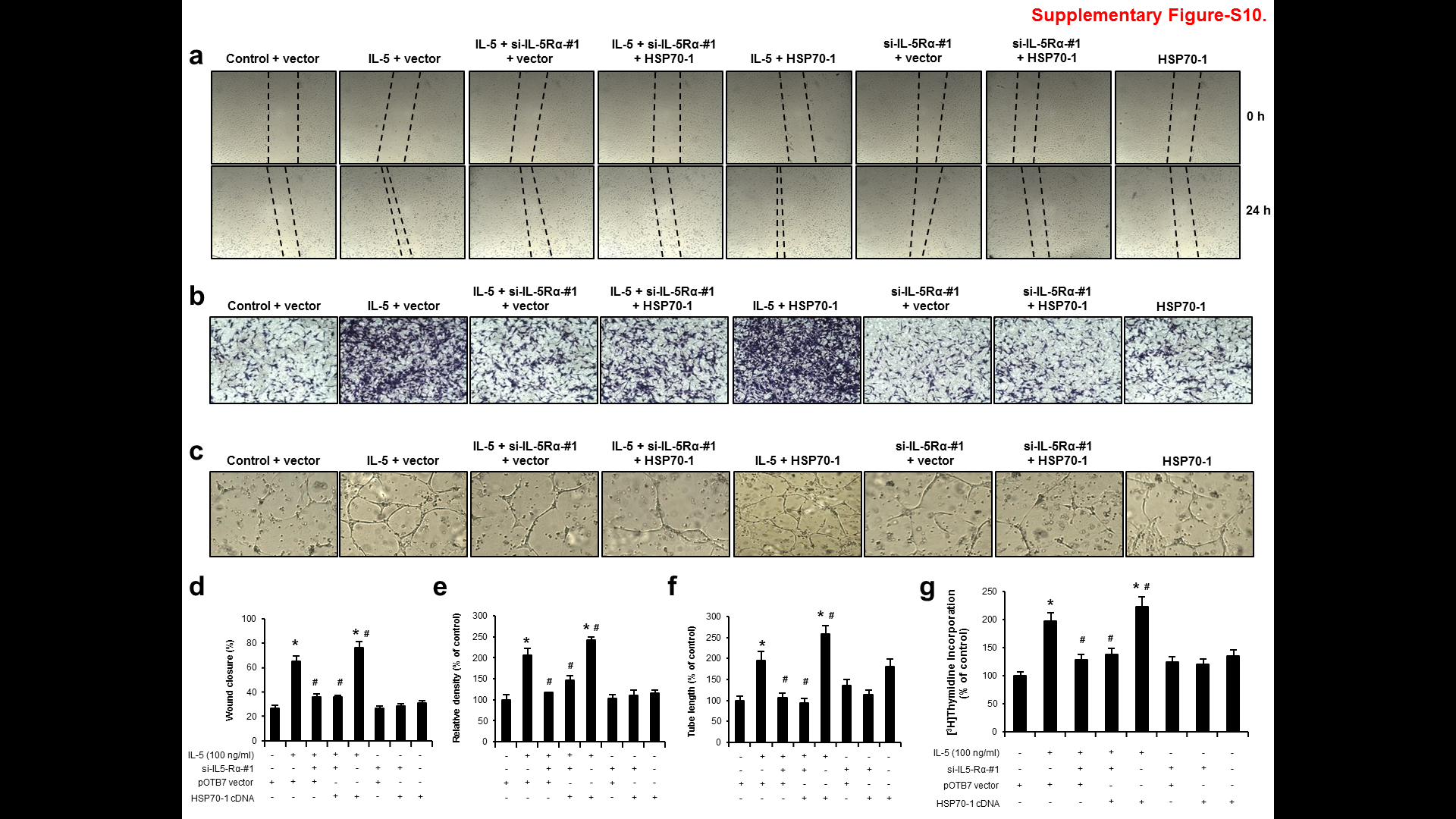


**Figure S10. Overexpression of HSP70-1 could not rescue the effect of IL-5Rαa-silencing on IL-5-induced endothelial cell proliferation, migration, invasion, and tube formation.** (**a-g**) After transfection of either HSP70-1 gene or IL-5Rα siRNA for 24 h, HUVECs were analyzed to evaluate the IL-5-stimulated proliferation, migration, invasion, and colony tube formation. All data are reported as the means ± SE from three independent experiments. **P* < 0.05 compared with control. ^#^*P* < 0.05 compared with IL-5 treatment.

**Supplementary Figure S11**


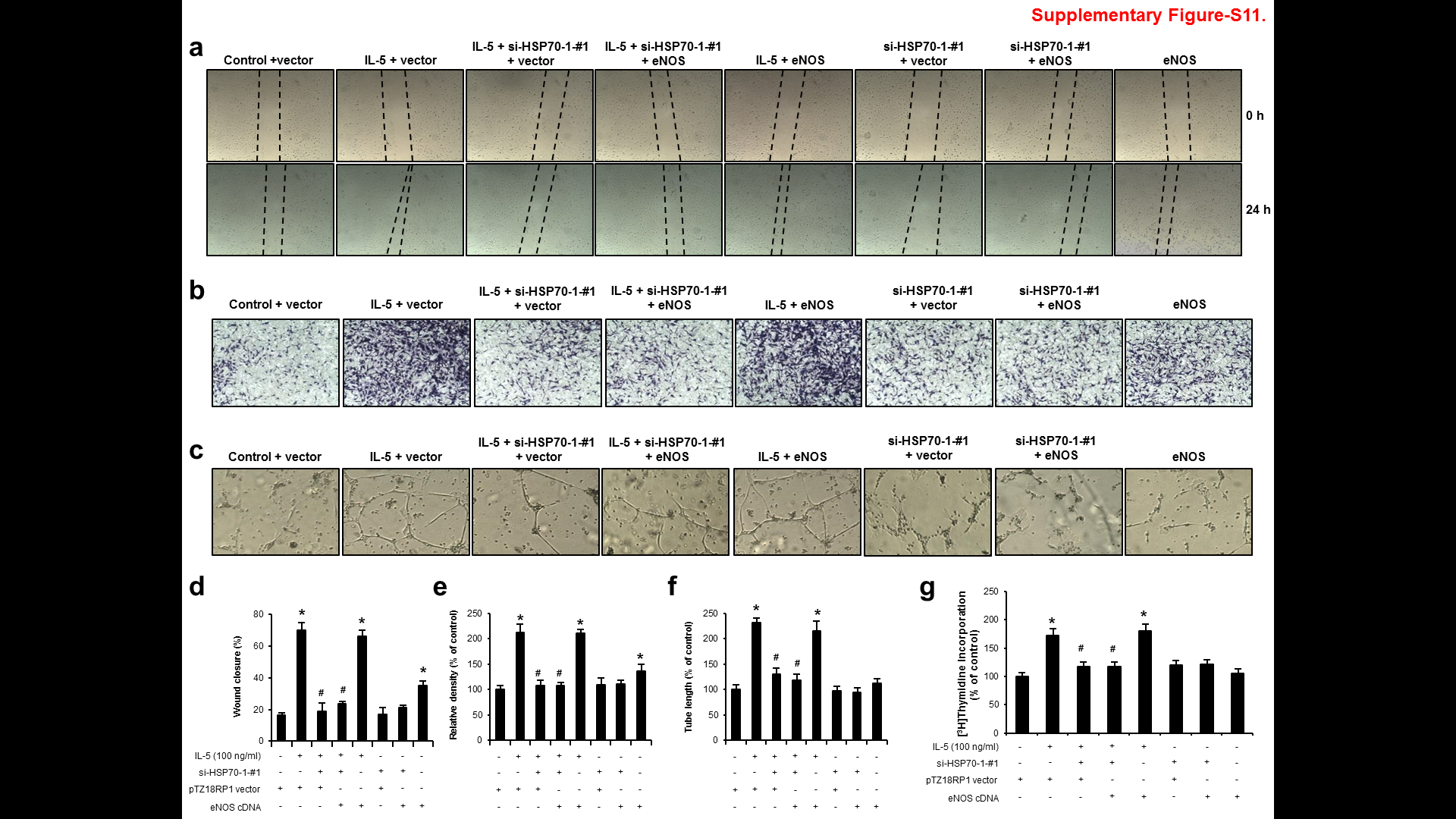


**Figure S11. Overexpression of the eNOS gene could not reverse the effect of HSP70-1 silencing on IL-5-induced endothelial cell proliferation, migration, invasion, and tube formation.** (**a-g**) After transfection of either eNOS gene or HSP70-1 siRNA for 24 h, HUVECs were analyzed to evaluate the proliferation, migration, invasion, and colony tube formation induced by IL-5. All data are reported as the means ± SE from three independent experiments. **P* < 0.05 compared with control, ^#^*P* < 0.05 compared with IL-5 treatment.

**Supplementary Figure S12**


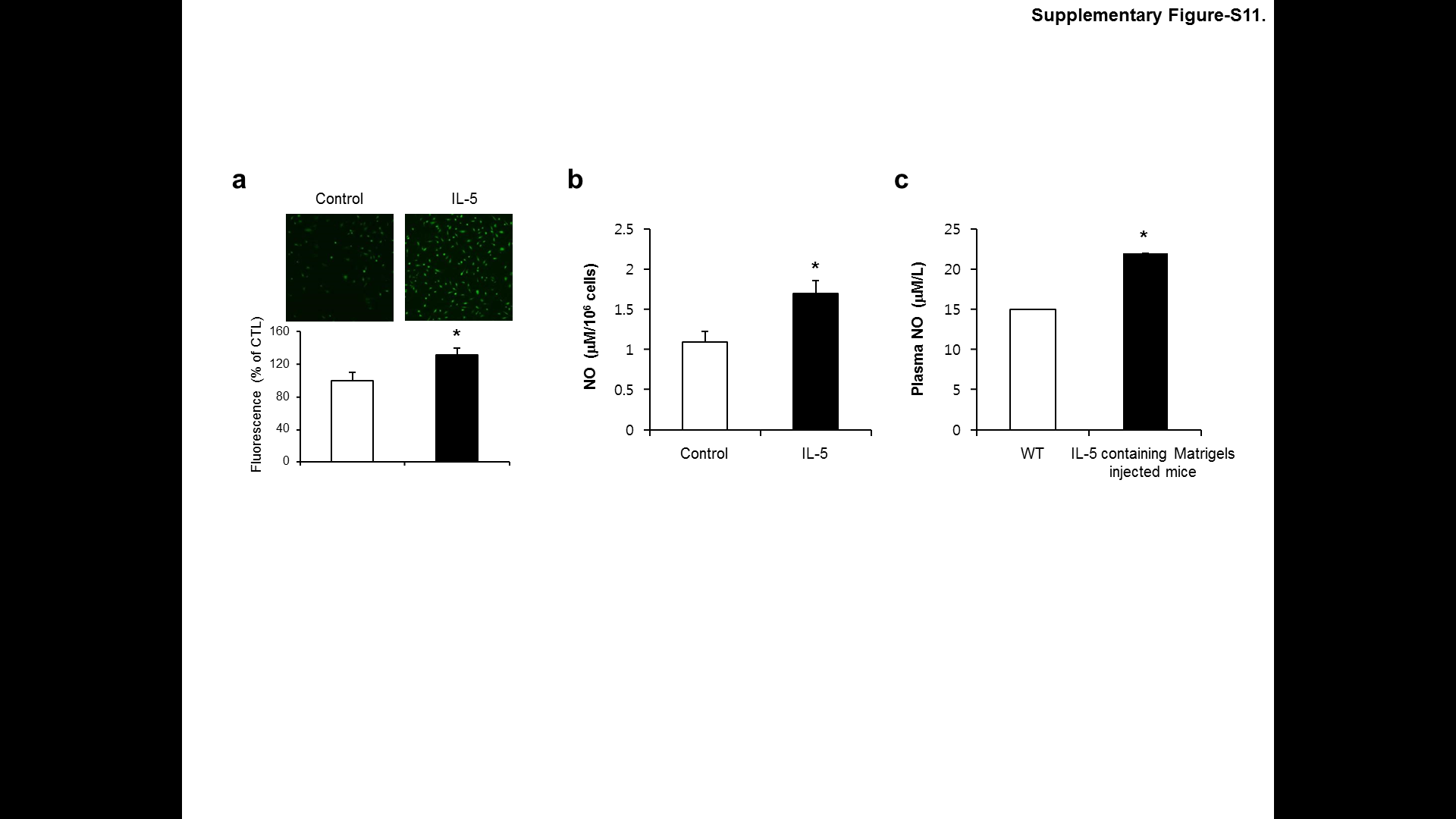


**Figure S12. IL-5 stimulated NO production in HUVECs and matrigel plug mice plasma.** (a) Intracellular NO production in IL-5-tretaed HUVECs. After addition of IL-5, HUVECs were incubated with DAF-FM, and the images were captured by a fluorescence microscope. Relative levels of intracellular NO production was analyzed with software device. (b) NO production in IL-5-treated HUVECs. HUVECs were treated with IL-5 for 6 h. Modified griess reaction was performed to detect NO production. (c) NO production was analyzed by a modified griess reaction in plasma from matrigel plug mice injected with IL-5.

All data are reported as the means ± SE from three independent experiments. **P* < 0.05 compared with control.

**Supplementary Figure S13 (1)**


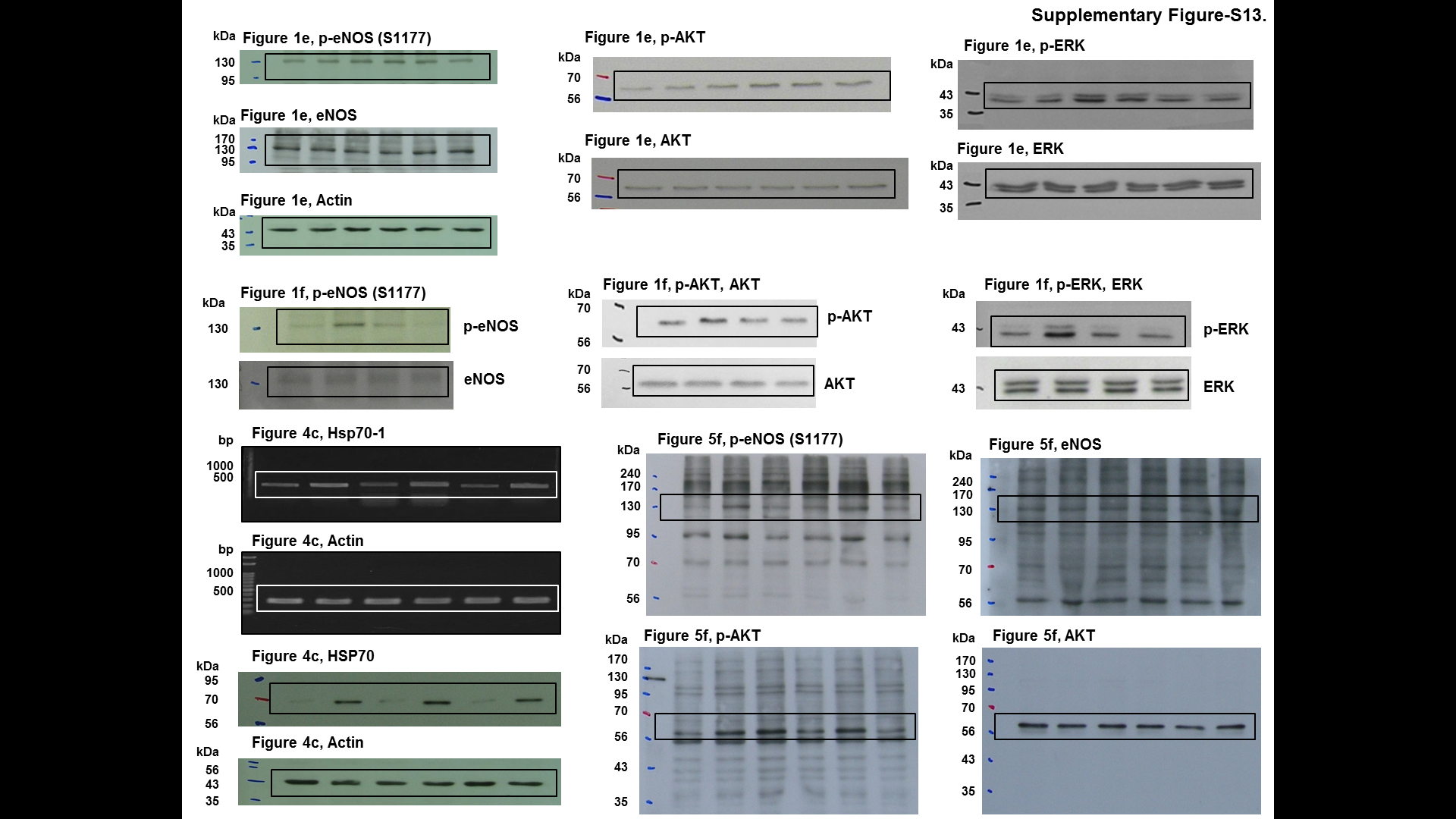


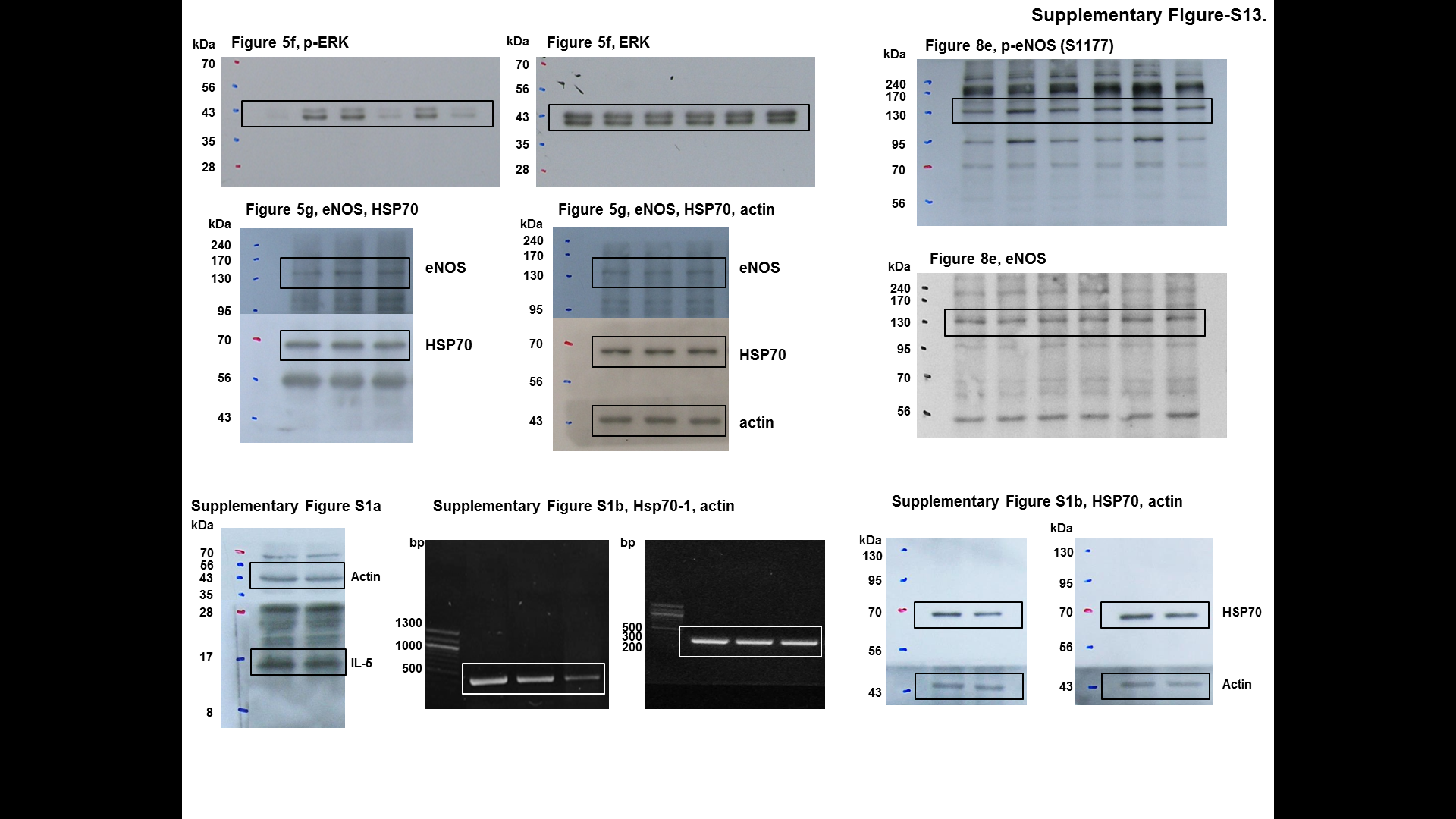


**Figure S13.** Uncropped immunoblots of the most important blots.

**Supplementary Figure S13 (2)**


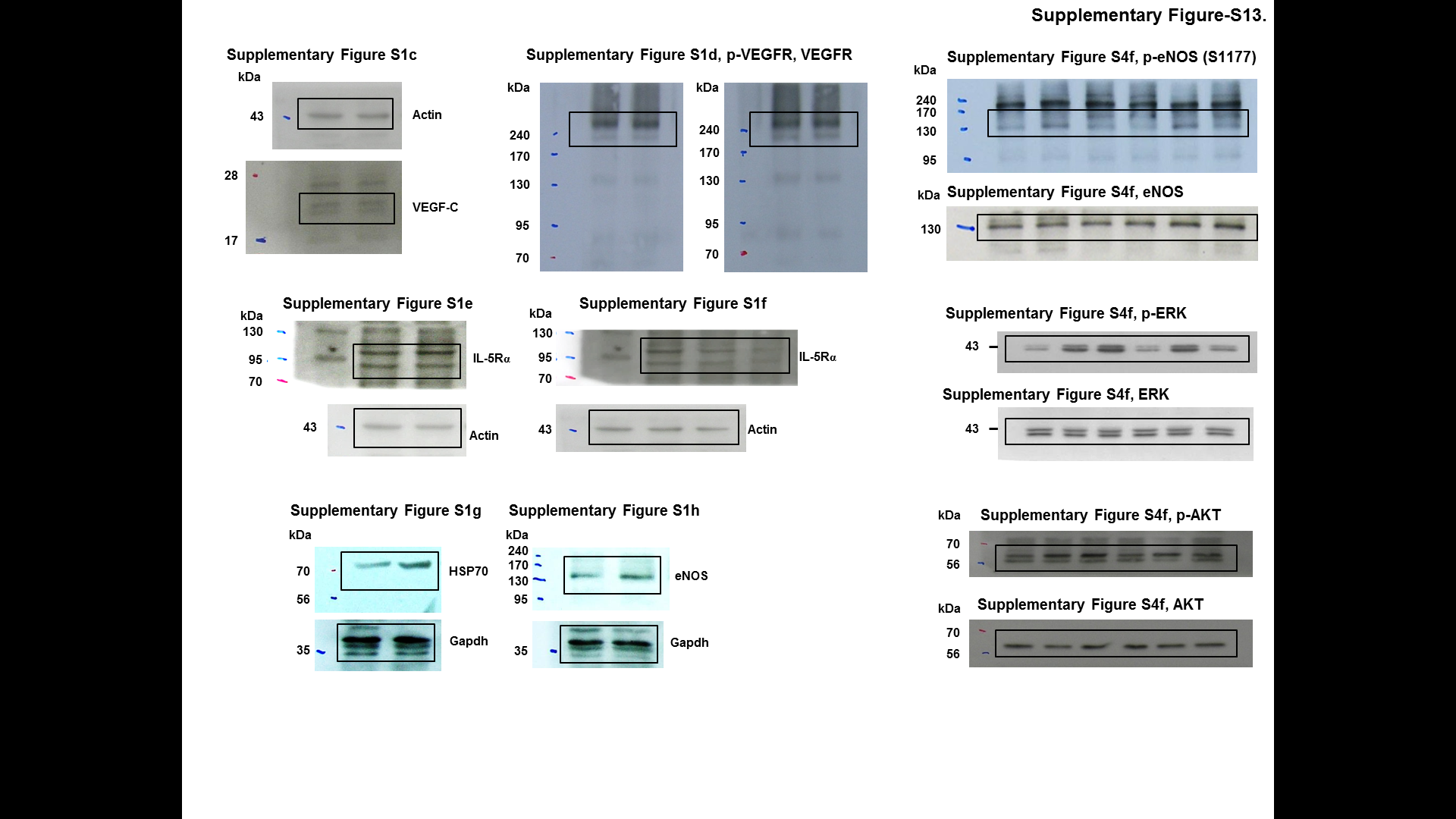


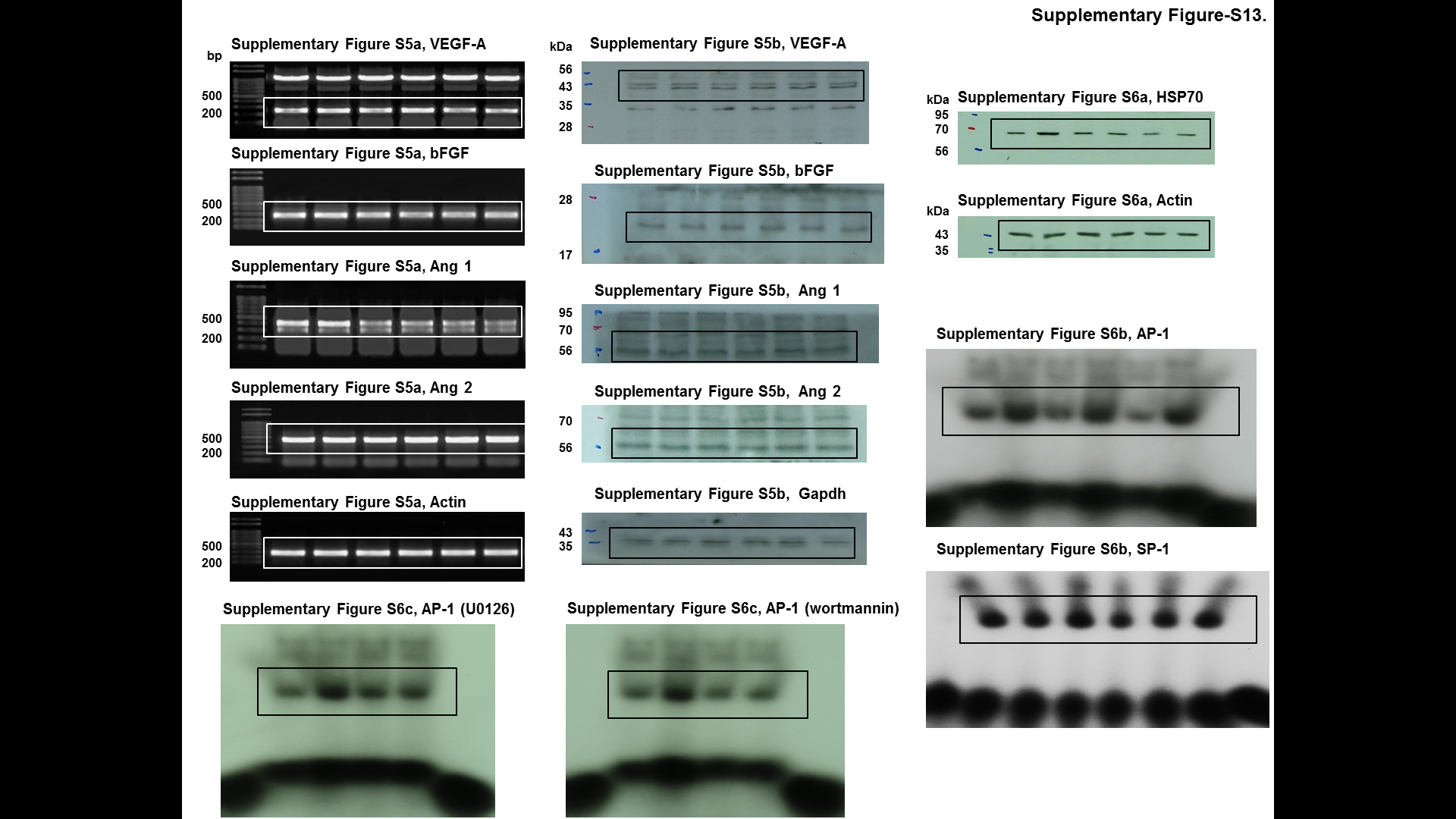


**Figure S13. Continued.**

**Supplementary Figure S13 (3)**


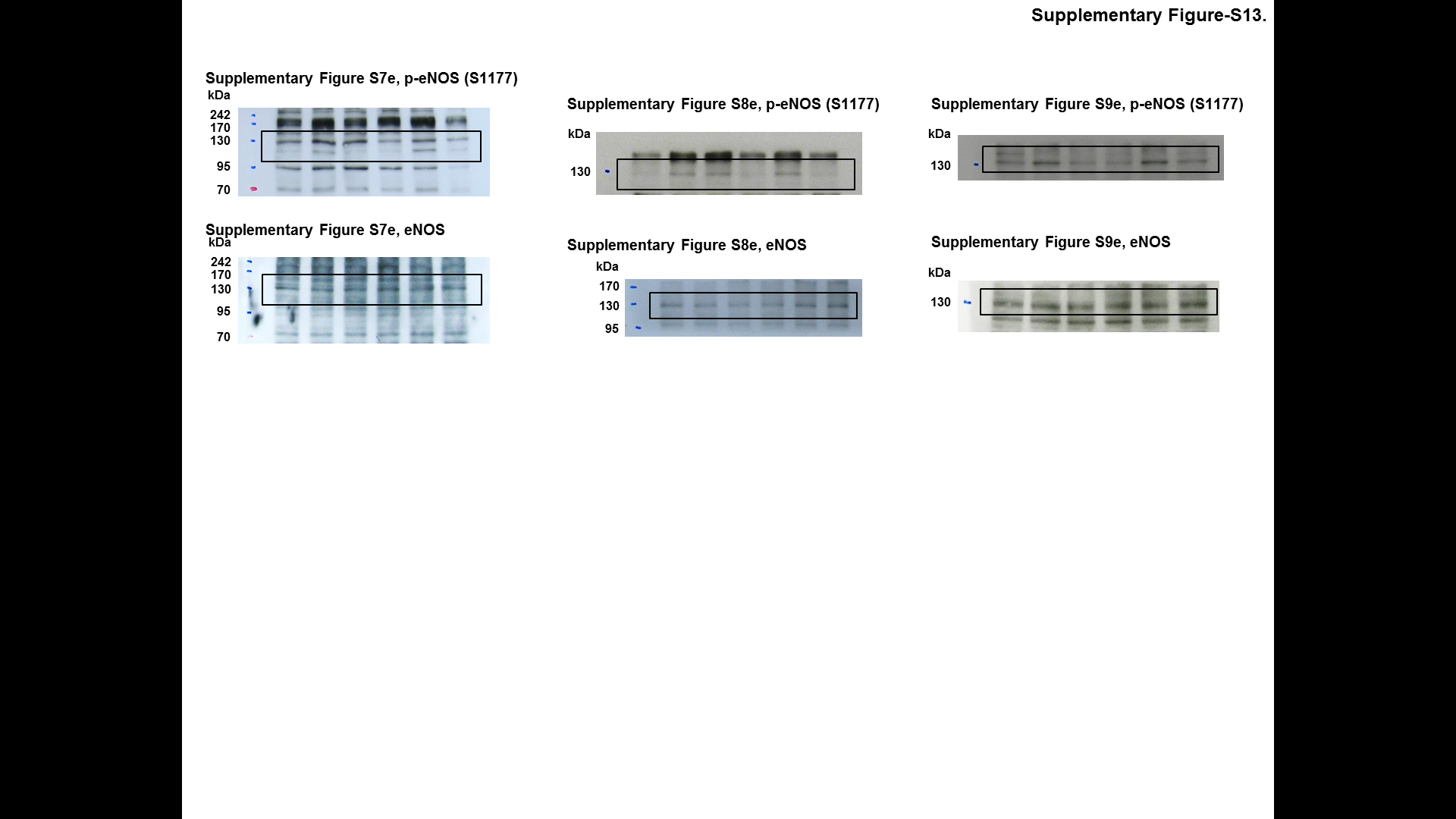


**Figure S13. Continued.**

**Supplementary Table**

**Table S1. Gene expression patterns in IL-5-treated HUVEC samples, compared to normal HUVEC samples at any time points.**

| **Gene Symbol** | **Genbank**  **Acc. No.** | **Fold change** | | |  | **Gene Symbol** | **Genbank**  **Acc. No.** | **Fold change** | | |
| --- | --- | --- | --- | --- | --- | --- | --- | --- | --- | --- |
|  |  | **6 h** | **12 h** | **24 h** |  |  |  | **6 h** | **12 h** | **24 h** |
| **LOC100131277** | XM_001716997.1 | 1.51 | 0.97 | 0.91 |  | **OSR2** | XM_001126824.1 | 0.98 | 0.94 | 0.65 |
| **PATL2** | XR_040922.1 | 1.55 | 0.96 | 0.88 |  | **DYRK2** | NM_006482.2 | 1.05 | 0.84 | 0.39 |
| **LOC100134006** | XM_001725030.1 | 1.51 | 0.95 | 0.85 |  | **LOC390349** | XM_372472.2 | 0.95 | 0.97 | 0.64 |
| **LOC100133797** | XM_001725482.1 | 1.52 | 0.94 | 0.80 |  | **DYSFIP1** | NM_001007533.3 | 1.04 | 1.08 | 0.65 |
| **SLMO2** | NM_016045.2 | 1.68 | 0.89 | 0.86 |  | **LOC100133005** | XM_001718200.1 | 0.85 | 0.64 | 0.88 |
| **HS.563862** | BU616091 | 1.51 | 0.92 | 0.88 |  | **KEAP1** | NM_203500.1 | 1.02 | 0.66 | 0.94 |
| **HS.545352** | BX103864 | 1.55 | 0.86 | 0.90 |  | **TAOK2** | NM_004783.2 | 0.94 | 0.64 | 1.01 |
| **ZNF114** | NM_153608.1 | 1.55 | 0.88 | 0.90 |  | **CTNNB1** | XM_001133675.1 | 0.97 | 0.63 | 0.97 |
| **MDK** | NM_001012334.1 | 1.56 | 0.83 | 0.83 |  | **LOC646010** | XM_933332.1 | 1.06 | 0.61 | 0.83 |
| **HS.545586** | BQ012728 | 1.57 | 0.87 | 0.85 |  | **HS.276854** | AI458759 | 1.09 | 0.63 | 0.87 |
| **RNY5** | NR_001571.2 | 1.57 | 0.86 | 0.86 |  | **HS.537828** | CA394309 | 1.14 | 0.66 | 1.07 |
| **BAHD1** | NM_014952.3 | 1.50 | 1.01 | 0.95 |  | **LOC152667** | NR_002228.1 | 1.26 | 0.66 | 0.86 |
| **HS.563340** | BM682355 | 1.51 | 1.01 | 0.99 |  | **LOC100132564** | XM_001713808.1 | 1.00 | 1.54 | 0.91 |
| **ARL9** | NM_206919.1 | 1.52 | 1.16 | 0.98 |  | **LYNX1** | NM_177476.2 | 0.86 | 1.54 | 0.97 |
| **LOC652456** | XM_941905.1 | 4.99 | 0.86 | 1.37 |  | **HSPE1** | NM_002157.1 | 0.88 | 1.57 | 1.23 |
| **HS.201858** | BX097814 | 1.56 | 0.95 | 1.07 |  | **EXOSC8** | NM_181503.1 | 0.75 | 2.01 | 1.22 |
| **HS.122288** | AA781928 | 1.51 | 1.00 | 1.11 |  | **GPR108** | NM_001080452.1 | 1.11 | 1.52 | 1.08 |
| **SIGLEC7** | NM_014385.2 | 1.58 | 1.04 | 1.09 |  | **LOC728253** | XM_001128502.2 | 0.52 | 1.05 | 1.20 |
| **LOC441511** | XM_497141.2 | 1.60 | 1.02 | 1.20 |  | **ATF3** | NM_001040619.1 | 0.49 | 1.07 | 1.25 |
| **CTSZ** | NM_001336.2 | 1.50 | 0.96 | 1.18 |  | **PTGS2** | NM_000963.1 | 0.65 | 1.04 | 1.15 |
| **DDX59** | NM_031306.2 | 1.54 | 0.87 | 1.03 |  | **HS.560292** | BX107348 | 0.66 | 1.07 | 1.17 |
| **KIFC1** | NM_002263.2 | 1.68 | 0.83 | 1.04 |  | **EGR1** | NM_001964.2 | 0.61 | 1.09 | 1.27 |
| **LOC388160** | XM_370894.4 | 1.64 | 0.86 | 1.12 |  | **TRIB3** | NM_021158.3 | 0.50 | 1.14 | 1.03 |
| **COL9A3** | NM_001853.3 | 1.57 | 1.25 | 1.17 |  | **CHAC1** | NM_024111.2 | 0.43 | 1.31 | 1.21 |
| **EDG1** | NM_001400.3 | 1.70 | 1.23 | 1.19 |  | **IL1A** | NM_000575.3 | 0.57 | 1.19 | 1.13 |
| **LOC100130562** | XM_001723702.1 | 1.09 | 0.99 | 1.55 |  | **STC2** | NM_003714.2 | 0.41 | 1.32 | 1.18 |
| **HSPA6** | NM_002155.3 | 1.07 | 0.91 | 3.06 |  | **PTGS2** | NM_000963.1 | 0.56 | 1.19 | 1.17 |
| **HSPA1A** | NM_005345.4 | 1.24 | 0.85 | 1.93 |  | **SLC7A5** | NM_003486.5 | 0.46 | 1.21 | 1.11 |
| **HSPA1B** | NM_005346.3 | 1.34 | 0.83 | 2.90 |  | **ADM2** | NM_024866.4 | 0.47 | 1.22 | 1.10 |
| **LOC645197** | XM_928234.2 | 1.02 | 0.88 | 1.51 |  | **CEBPB** | NM_005194.2 | 0.53 | 1.15 | 1.12 |
| **SDC1** | NM_002997.4 | 1.17 | 1.05 | 1.59 |  | **DDIT3** | NM_004083.4 | 0.40 | 1.24 | 1.21 |
| **DCST1** | NM_152494.1 | 0.95 | 1.02 | 1.58 |  | **DDIT4** | NM_019058.2 | 0.56 | 1.11 | 1.14 |
| **FAM69B** | XM_001130258.1 | 0.98 | 1.01 | 1.52 |  | **HS.184721** | AW954199 | 0.64 | 1.26 | 1.03 |
| **GABRE** | NM_004961.3 | 1.30 | 0.89 | 1.52 |  | **HS.563969** | BM986974 | 0.62 | 0.94 | 0.96 |
| **HS.444587** | AI694603 | 1.26 | 1.54 | 1.57 |  | **FOXP1** | NM_032682.4 | 0.66 | 0.95 | 0.98 |
| **LOC100134266** | XR_037131.1 | 1.05 | 0.88 | 0.63 |  | **IL8** | NM_000584.2 | 0.64 | 1.09 | 0.94 |
| **LOC652094** | XM_945200.1 | 1.15 | 0.84 | 0.64 |  | **IL8** | NM_000584.2 | 0.56 | 1.09 | 0.90 |
| **ROBO2** | NM_002942.1 | 0.96 | 0.87 | 0.63 |  | **LOC100128485** | XM_001717043.1 | 0.61 | 1.18 | 0.78 |
